# The *Nicotiana tabacum L. major latex protein-like protein 423* (*NtMLP423*) positively regulates drought tolerance by ABA-dependent pathway

**DOI:** 10.1186/s12870-020-02690-z

**Published:** 2020-10-16

**Authors:** Heng Liu, Xiaocen Ma, Shaohua Liu, Bingyang Du, Nini Cheng, Yong Wang, Yuanhu Zhang

**Affiliations:** 1 State Key Laboratory of Crop Biology, College of Life Sciences, Shandong Agricultural University, Taian, Shandong 271018 P.R. China; 2 Linyi University, Linyi, 276005 Shandong P.R. China

**Keywords:** *NtMLP423*, Drought stress, ABA, *Nicotiana tabacum*

## Abstract

**Background:**

Drought stress is an environmental factor that limits plant growth and reproduction. Little research has been conducted to investigate the *MLP* gene in tobacco. Here, *NtMLP423* was isolated and identified, and its role in drought stress was studied.

**Results:**

Overexpression of *NtMLP423* improved tolerance to drought stress in tobacco, as determined by physiological analyses of water loss efficiency, reactive oxygen species levels, malondialdehyde content, and levels of osmotic regulatory substances. Overexpression of *NtMLP423* in transgenic plants led to greater sensitivity to abscisic acid (ABA)-mediated seed germination and ABA-induced stomatal closure. *NtMLP423* also regulated drought tolerance by increasing the levels of ABA under conditions of drought stress. Our study showed that the transcription level of ABA synthetic genes also increased. Overexpression of *NtMLP423* reduced membrane damage and ROS accumulation and increased the expression of stress-related genes under drought stress. We also found that *NtWRKY71* regulated the transcription of *NtMLP423* to improve drought tolerance.

**Conclusions:**

Our results indicated that *NtMLP423*-overexpressing increased drought tolerance in tobacco via the ABA pathway.

## Background

Drought stress is not conducive to plant growth and development, as it can cause changes in plant morphology and damage to cells [1, 2]. Plants have evolved many complex physiological and biochemical mechanisms to adapt to drought. The plant hormone, abscisic acid, regulates the physiological processes of plants under biotic and abiotic stresses [3].

Abscisic acid is a key sesquiterpene which is participated in many important processes of plant growth and development, and controls many genes related to stress adaptation responses and osmotic adjustment [4–6]. The increase in ABA synthesis under drought stress can promote stomatal closure and reduce transpiration loss [7]. Due to the role of ABA in response to drought stress, genes involved in the biosynthesis of ABA have been identified, such as 9-cis-epoxycarotenoid dioxygenase (*NCED*), xanthoxin dehydrogenase/reductase (*ABA2*), and ABA-aldehyde oxidase 3 (*AAO3*) [8, 9]. In Arabidopsis, *NCED3* contributes to ABA accumulation in response to drought stress [10], while the *aba2* and *aao3* mutants decreased ABA levels under drought stress [11, 12].

The major latex protein (*MLP*) gene was first identified in latex of the opium poppy [13, 14]. Orthologs of MLP, called MLP-like proteins, were found in other plants [15, 16]. Each plant species can contain multiple members of the MLP family. For example, there are 26, 14, and 27 *MLPs* in *Arabidopsis*, *Vitis vinifera*, and *Solanum lycopersicum*, respectively [17, 18]. The MLP family is characterized by low sequence similarities, whereas the three-dimensional structures are similar. It was found that two *MLPs* in Arabidopsis could delay flowering by inducing cis-cinnamic acid [19] and that *MLP* is closely related to ripening in fruits such as peach and kiwifruit [20]. Overexpression of *GhMLP* increases the flavonoid content of *Arabidopsis* and increases tolerance to salt stress [21], while the *MLP* expression level in wild strawberry and cucumber is increased by mechanical damage [22]. The expression of *MLP* gene family differs significantly in different tissues; however, there is little research on *MLP* genes, and the molecular mechanism of *MLPs* in response to abiotic stress remains elusive in tobacco. Here, we cloned the *NtMLP423* gene from tobacco and tested the stress response of *NtMLP423*-overexpressing transgenic plants.

## Results

### Subcellular localization of NtMLP423

We constructed the Pro35S::MLP423-GFP vector and injected it into the leaves of *Nicotiana benthamiana* by agroinfiltration. Confocal microscopy results indicated that NtMLP423 protein was localized in cytoplasm and nucleus (Fig. S1).

### Expression analysis of *NtMLP423* in tobacco

**Fig. 1:**
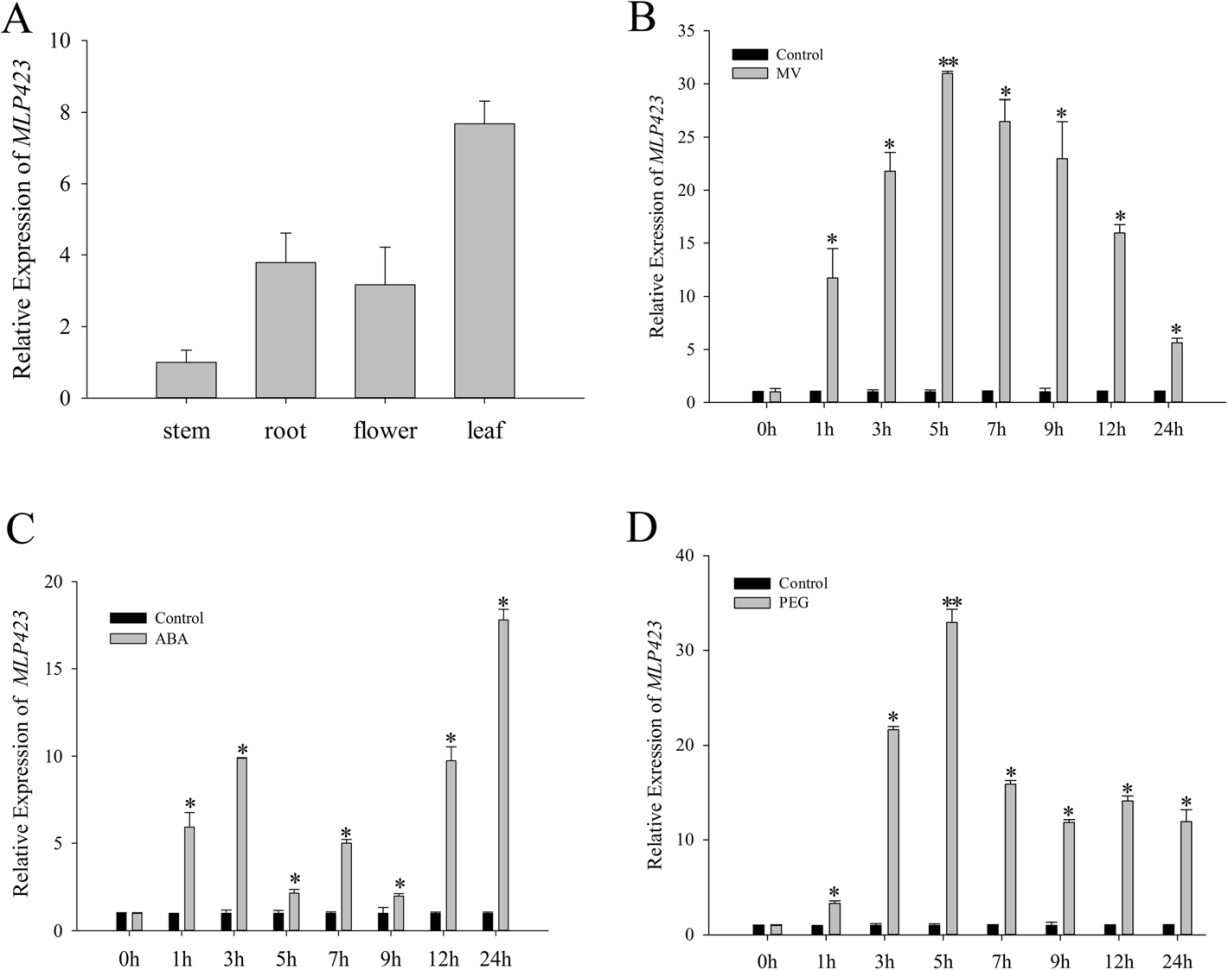
Expression analysis of *NtMLP423*. Expression of *NtMLP423* gene in different tissues (**a**), under 10 μM MV treatments in tobacco leaves (**b**), under 100 μM ABA treatments in tobacco leaves (**c**), and under 20% PEG treatments in tobacco leaves (**d**). Data represent means ± SE (*n* = 3). * indicate significant difference relative to controls (*P < 0.05, **P < 0.01)

The tissue expression analysis of *NtMLP423* by qRT-PCR showed the highest expression of *NtMLP423* in the leaves, followed by the roots, with the lowest expression in the stems (Fig. 1a). Expression of *NtMLP423* under methyl viologen (MV), ABA and polyethyleneglycol (PEG) treatments were detected by qPCR. Results indicated differential upregulation under the three treatments (Fig. 1b-d). The induction of *ABI5* (abscisic acid insensitive 5; ABA-responsive gene) [23], *P5CS* (pyrroline-5-carboxylate synthase; proline biosynthesis key gene), and *DEFL* (defensin-like; H_2_O_2_ and MV-responsive gene) [24, 25] ensured that the treatments were effective (Fig. S2). The induction rate in the PEG treatment was more than 30 times higher than that in control group over 5 h, while that in the ABA treatment for 24 h was the most significant. These results suggested that *NtMLP423* was regulated by drought stress and drought-related signaling molecules.

### Overexpression of *NtMLP423* confers drought tolerance in *Arabidopsis*

**Fig. 2:**
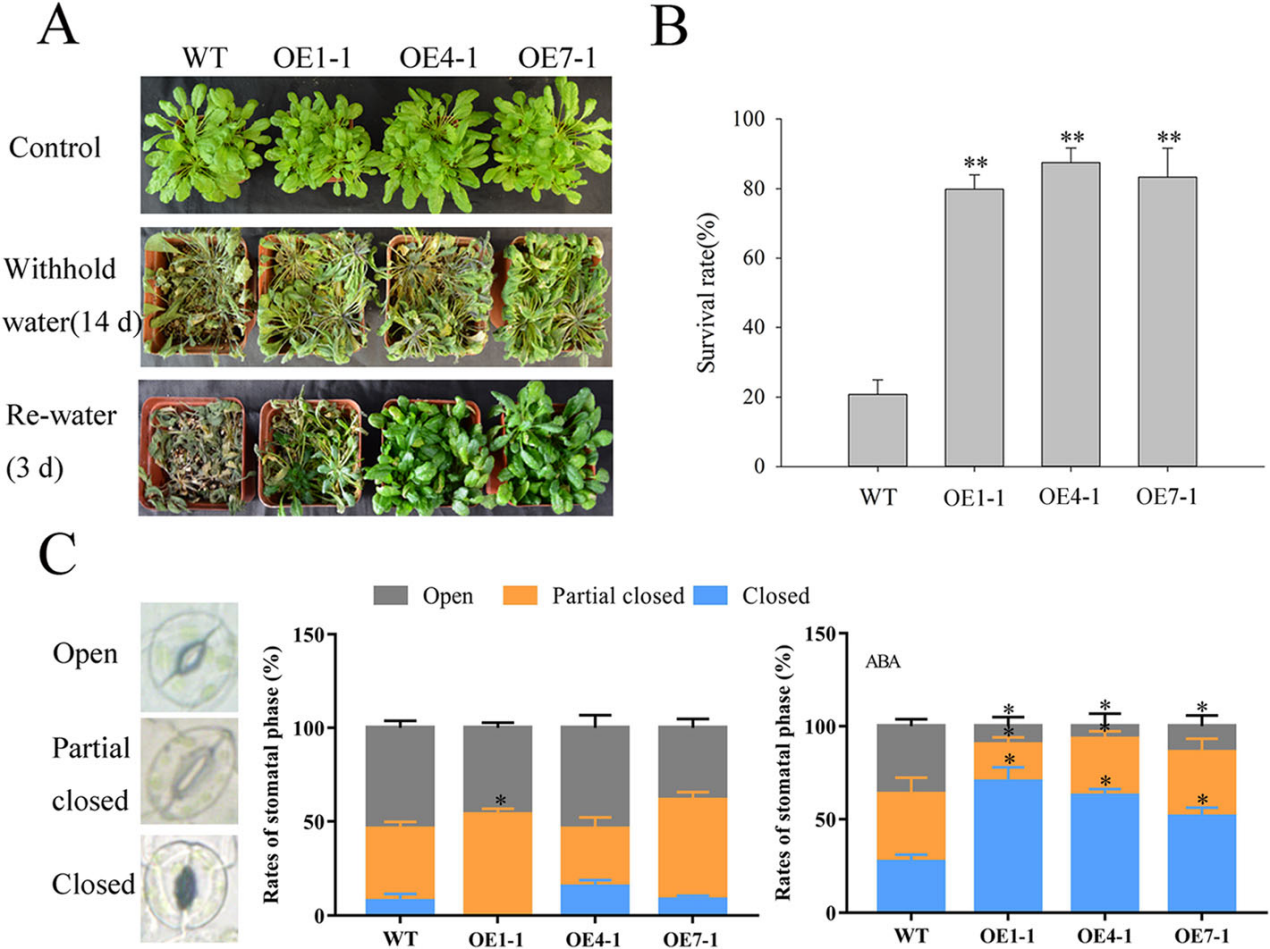
The *NtMLP423* gene is involved in drought stress responses in Arabidopsis. **a** Irrigation was discontinued for 14 days, followed by 3 days of watering and observation of phenotypes after re-watering. **b** Survival rate of plants treated with drought stress. **c** Stomatal aperture after ABA treatment. The proportions of different stomata phases were calculated with or without ABA treatment. Data are means ± SE (n = 3). * indicate significant difference (*P < 0.05, **P < 0.01)

To investigate whether *NtMLP423* is participated in drought stress, we obtained transgenic Arabidopsis expressing the *NtMLP423* gene. *NtMLP423* was expressed in all transgenic Arabidopsis (Fig. S3), and three T3-generation homozygous lines (OE1–1, OE4–1 and OE7–1) were selected for subsequent experiments. Transgenic and wild type (WT) seeds were sown in murashige and skoog (MS) medium and in a mannitol medium. The results suggested that there was no difference in germination rate in MS medium; however, germination rate of overexpressing *NtMLP423* Arabidopsis were higher than that of WT seeds in different concentrations of the mannitol medium (Fig. S4A, B). The average root length of overexpressing *NtMLP423* plants was significantly longer than that of WT plants (Fig. S4C, D). We examined the effects of *NtMLP423* overexpression under drought stress by discontinuing irrigation to the plants for 2 weeks, followed by a watering period of 3 d to observe the recovery process. The results showed that only 20.8% of WT plants survived after watering resumed, whereas transgenic survival rate was more than 80% (Fig. 2a-b). We further examined the effects of *NtMLP423* overexpression under drought stress by treating plants with 20% PEG. We found that WT plants had more severe wilting than the transgenic plants (Fig. S5A). Relative water content of transgenic plants were found to be much higher than that of WT following drought stress (Fig. S5B). Osmotic potential of leaves of overexpressing *NtMLP423* were also significantly lower than that of WT under drought stress (Fig. S5C). The results suggested that overexpression of *NtMLP423* increased the resistance to drought stress in Arabidopsis.

### Overexpression of *NtMLP423* increases ABA sensitivity and accumulation in Arabidopsis

The ABA hormone plays a key role in regulating growth and responses to various stress conditions in plants, including stomatal closure [26]. We detected ABA-induced stomatal closure (pore phase was defined as closed, partially open, or open, based on the ratio of width to length of the pore diameter). There were no significant differences in ratios between all lines without ABA treatment. However, after ABA treatment for 30 min, the percentage of stomatal closure in overexpressing *NtMLP423* was much higher than in WT (Fig. 2c). The percentage of stomatal closure of WT, OE1–1, OE4–1 and OE7–1 transgenic plants increased by 2.56 times, 22.67 times, 3.52 times, and 6.22 times, respectively, after ABA treatment. The results suggested that the improvement of drought tolerance is associated with ABA-induced stomatal closure.

**Fig. 3:**
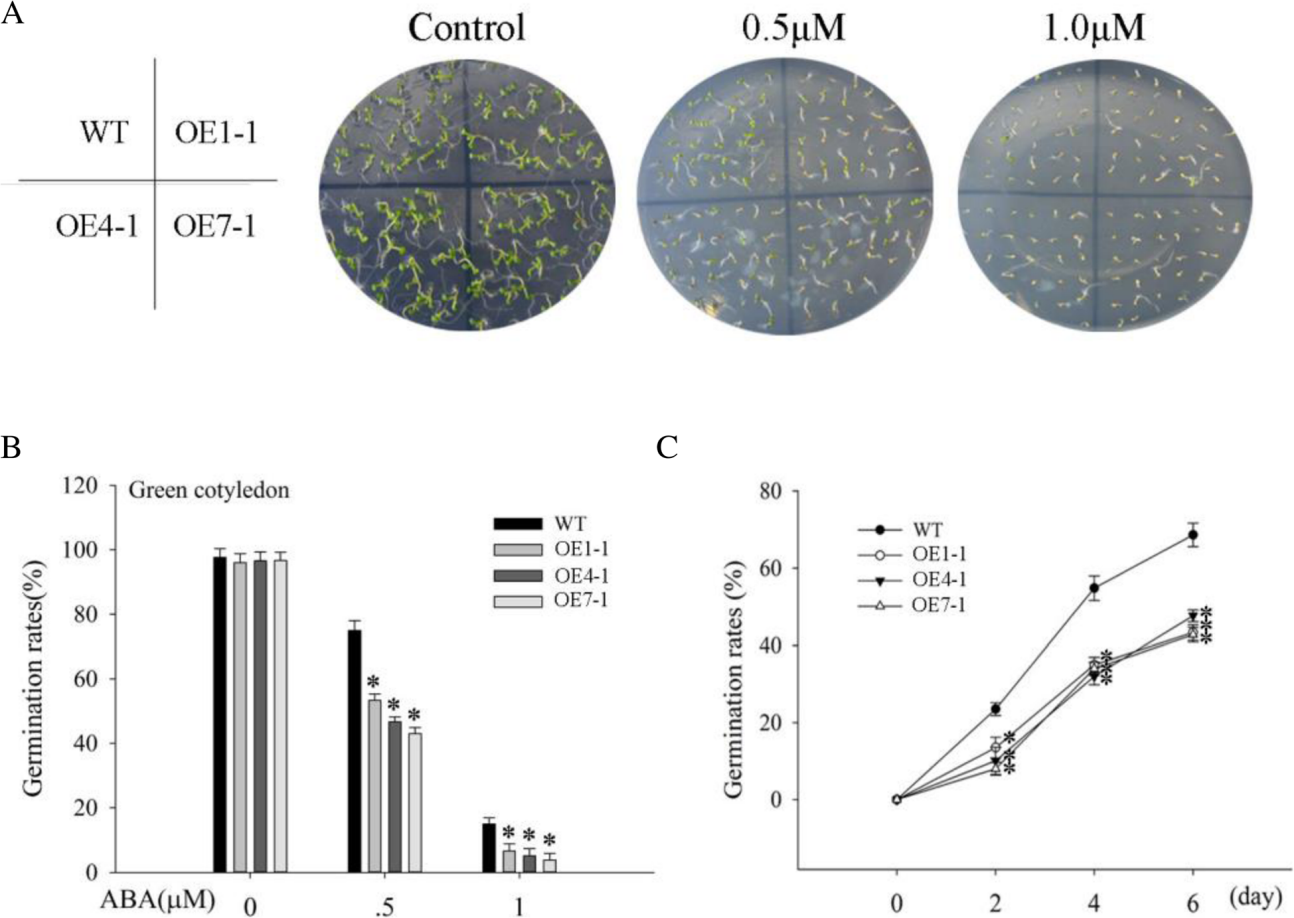
*NtMLP423* gene involvement in ABA responses in the seed germination assay. **a** Seeds growing on MS medium added with ABA. **b** Germination rates under ABA treatment. **c** Germination rate changes with time under 0.5 μM ABA. Data represent means ± SE (n = 3). * indicate significant difference relative to WT (*P < 0.05)

**Fig. 4:**
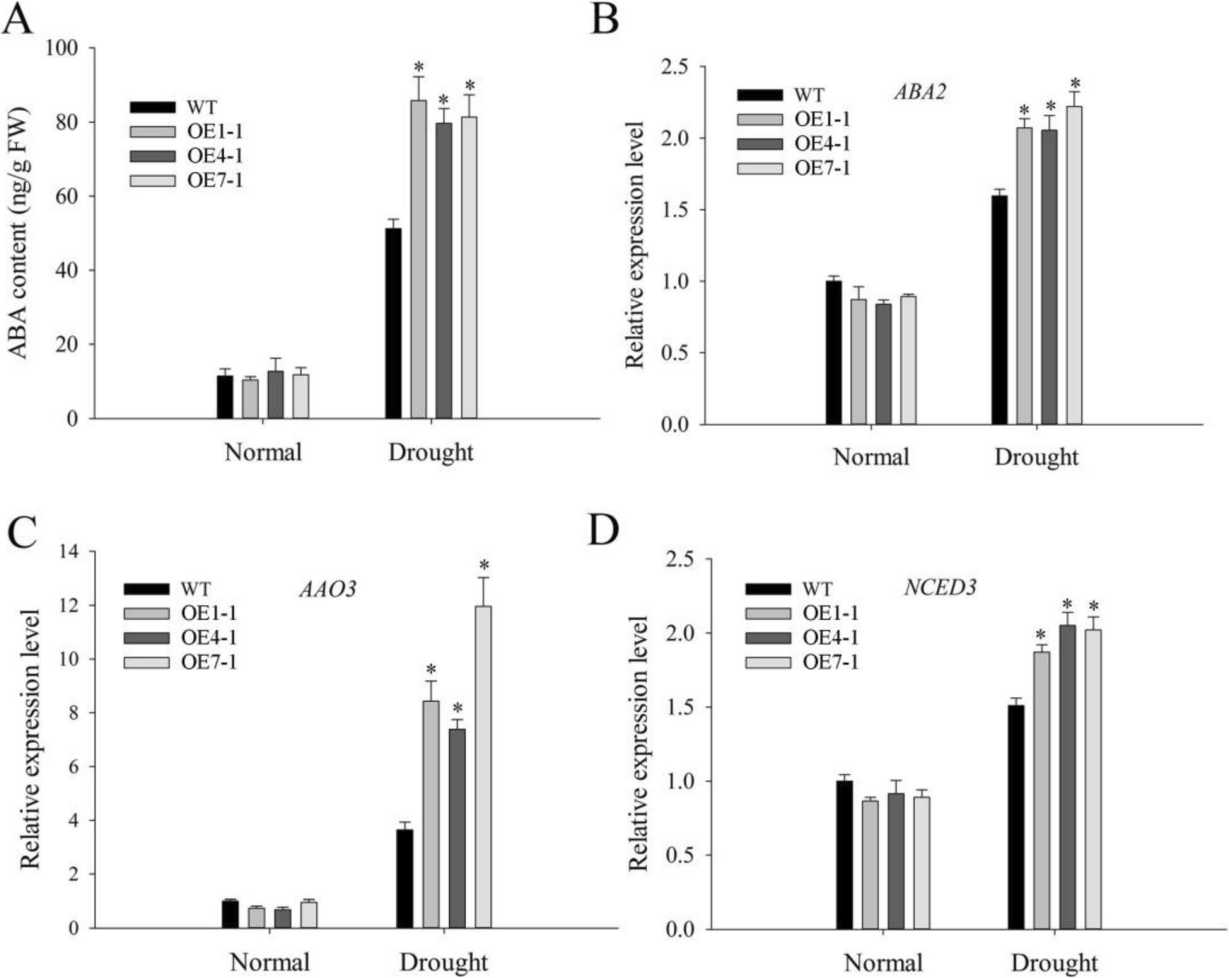
ABA content in Arabidopsis leaves and gene expression related to ABA biosynthesis under drought stress. **a** ABA content under normal (untreated) conditions and after 20% PEG treatment. **b**-**d** Expression levels of *ABA2*, *AAO3*, and *NCED3* under drought stress. Data represent means ± SE (n = 3). * indicate significant difference relative to WT (*P < 0.05)

To evaluate the role of ABA as a signal molecule during plant stress response [3], we conducted ABA treatments. In the medium without ABA, there were no differences in germination rates among all plants. At different ABA concentrations (0.5 and 1.0 μM), plants in which *NtMLP423* was overexpressed indicated significantly decreased germination rates compared to WT plants (Fig. 3), indicating that overexpression of *NtMLP423* improves ABA sensitivity. Under normal conditions, ABA content in all plants was similar, while it was significantly higher in overexpressing *NtMLP423* than in WT plants under drought stress, which increased by approximately 2.0 times (Fig. 4a). We further measured the expression levels of ABA-related genes and found that expression levels of ABA-synthesizing genes (*ABA2, AAO3,* and *NCED3*) in overexpressing *NtMLP423* were significantly higher than those in WT under drought conditions (Fig. 4b-d). Here, we also tested the genes involved in ABA catabolism pathway (Fig. S6). The results indicated that expression of the *BG1* (β-glucosidase 1) gene in overexpressing *NtMLP423* plants were significantly higher than that of WT under drought stress, but the expression level of *CYP707A1* (cytochrome P450 monooxygenase 707A1) gene was lower than that of WT under drought stress (Fig. S6A, S6C). *BG2* (β-glucosidase 2) and *UGT71C5* (UDP-glucosyltransferase 71C5) gene expressions were not significantly different in all lines that were subjected to drought stress (Fig. S6B, S6D).

### Overexpression of *NtMLP423* confers drought tolerance in tobacco

**Fig. 5:**
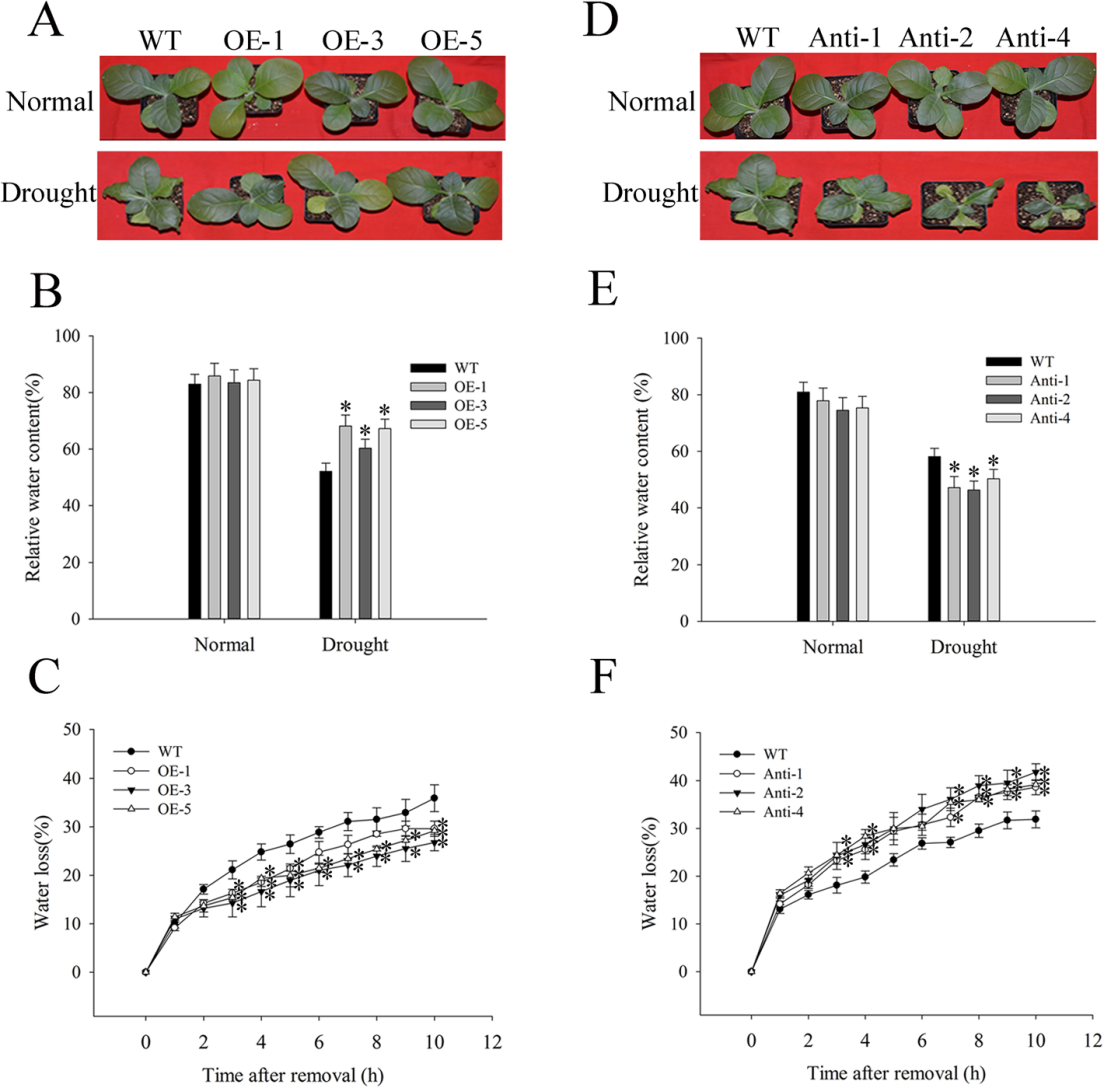
Overexpression of *NtMLP423* enhances drought tolerance in tobacco. **a** Phenotypic observation of overexpressing *NtMLP423* plants treated with 20% PEG for 7 days. **b** RWC of overexpressing *NtMLP423* plants. **c** Water loss rates of overexpressing *NtMLP423* detached leaves. Tobacco plants carrying antisense *NtMLP423* gene showed increased sensitivity to drought stress. **d** Phenotypic observation of antisense transgenic plants treated with 20% PEG for 7 days. **e** RWC of antisense transgenic plants. **f** Water loss rates of antisense transgenic detached leaves. Data represent means ± SE (n = 3). * indicate significant difference relative to WT (*P < 0.05)

To examine drought tolerance in tobacco, we detected the expression level of *NtMLP423* in transgenic lines and selected three lines of the T2-generation overexpressing *NtMLP423* (OE-1, OE-3, and OE-5), and three antisense transgenic lines (Anti-1, Anti-2, and Anti-4) for subsequent analysis (Fig. S7). We examined the effects of *NtMLP423* overexpression in tobacco under drought stress using 20% PEG treatments. We found more severe wilting in WT than in overexpressing *NtMLP423* plants (Fig. 5a). RWC of overexpressed plants was much higher than that of WT after drought stress and RWC of OE-1, OE-3, and OE-5 increased by 1.17, 1.11, and 1.14 times, respectively, compared to WT. The water loss rate of overexpressing *NtMLP423* leaves in vitro was lower than that of WT leaves (Fig. 5b-c). However, antisense transgenic tobacco showed the opposite result, which reduces the resistance to drought stress, as shown in Fig. 5d-f. Antisense plants had more severe wilting than the WT, and RWC was lower than that of the WT; however, the rate of water loss from antisense plants was higher than that of WT (Fig. 5d-f). In addition, the osmotic potential of tobacco leaves were measured and found that osmotic potential of overexpressing *NtMLP423* was lower than that of WT, while antisense plants had the highest osmotic potential (Fig. S8). The results indicated that *NtMLP423* overexpression increased resistance to drought stress in tobacco.

### Overexpression of *NtMLP423* enhances photosynthesis under drought stress in tobacco

**Fig. 6:**
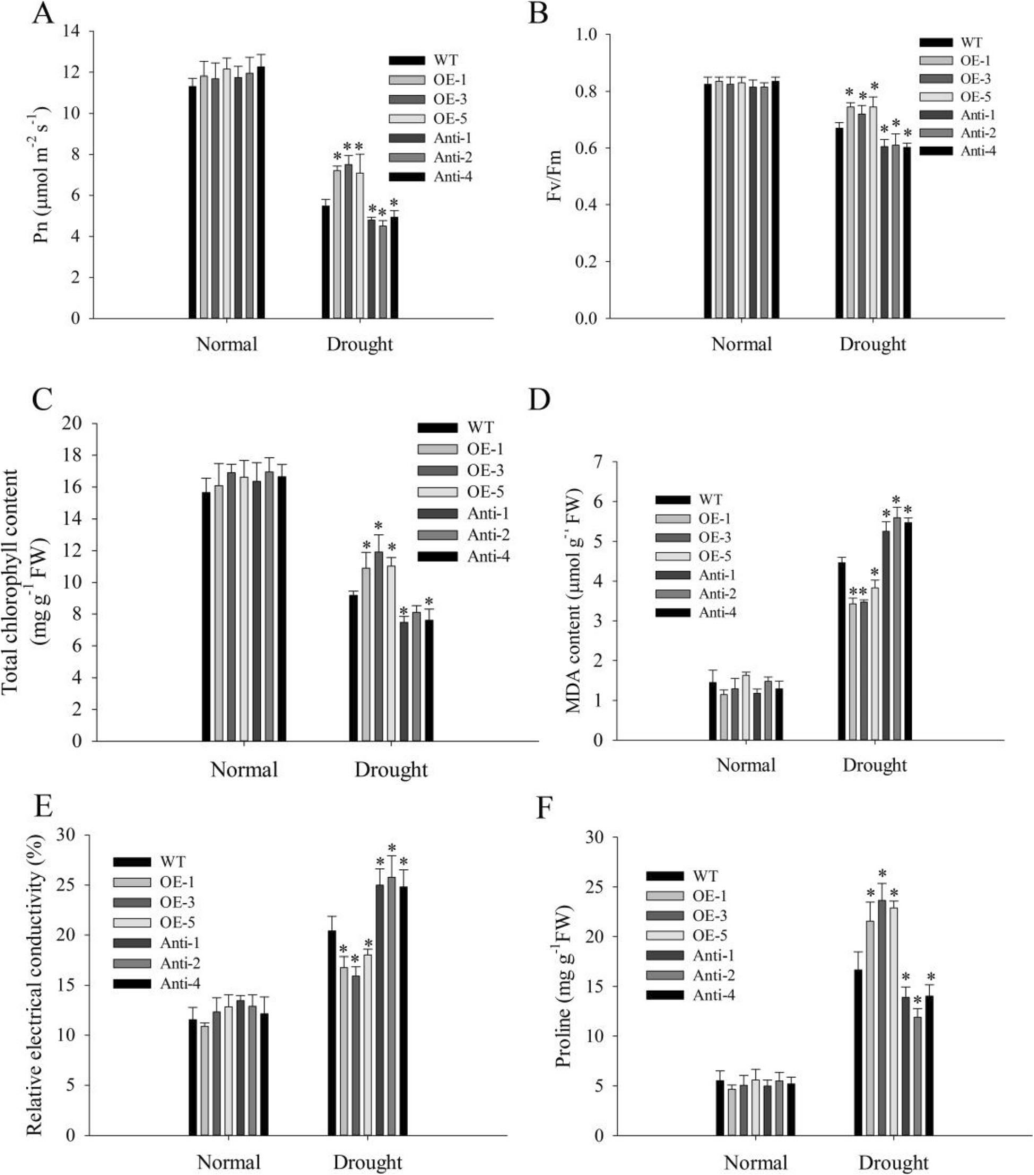
Physiological determination of tobacco under drought stress. **a** Pn of tobacco leaves. **b** Fv/Fm ratio of tobacco. **c** Chlorophyll content determination. **d** MDA content of plants. **e** Relative electrical conductivity in plants. **f** Proline content under drought stress. Data represent means ± SE (n = 3). * indicate significant difference relative to WT (*P < 0.05)

Net photosynthetic rates (Pn) of all plants decreased markedly after drought stress, with the greatest decrease in the antisense line and the lowest decrease in the overexpressed line. The Pn of WT, OE-1, OE-3, OE-5, Anti-1, Anti-2, and Anti-4 decreased by 47.3, 33.8, 32.5, 41.2, 62.7, 63.1, and 63.3%, respectively, after drought stress treatment (Fig. 6a). The variable fluorescence/maximum fluorescence (Fv/Fm) ratio of all plants decreased after drought stress. However, the decrease in Fv/Fm of antisense was larger than that of other lines, while the decrease in Fv/Fm of overexpressed plants was smaller (Fig. 6b). Chlorophyll content of all lines decreased markedly, by 41.1, 37.5, 29.5, 33.7, 54.2, 52.3, and 54.3%, in WT, OE-1, OE-3, OE-5, Anti-1, Anti-2, and Anti-4, respectively (Fig. 6c).

### Overexpression of *NtMLP423* alleviates membrane damage and reduces ROS levels under drought stress in tobacco

The cell membrane of plants is subject to damage under adverse conditions, the extent of which can be reflected by levels of malondialdehyde (MDA). MDA content increased in all groups under drought stress; however, MDA content in overexpressed was lower than that in WT plants, and antisense plants showed the highest content (Fig. 6d). In addition, the relative electrical conductivity of overexpressing *NtMLP423* was the lowest, while that of antisense plants was the highest after drought stress (Fig. 6e). These results showed that membrane damage in overexpressed plants was the lowest under drought stress. Because proline is a key intracellular osmotic regulator and plays an important role in osmotic stress resistance [27], proline content in the different plant lines were measured. Drought stress improved proline content in all lines, with the highest content in overexpressed plants and the lowest in antisense plants (Fig. 6f). The results indicated that overexpressing plants could reduce osmotic damage and membrane damage under drought stress by regulating content of proline.

**Fig. 7:**
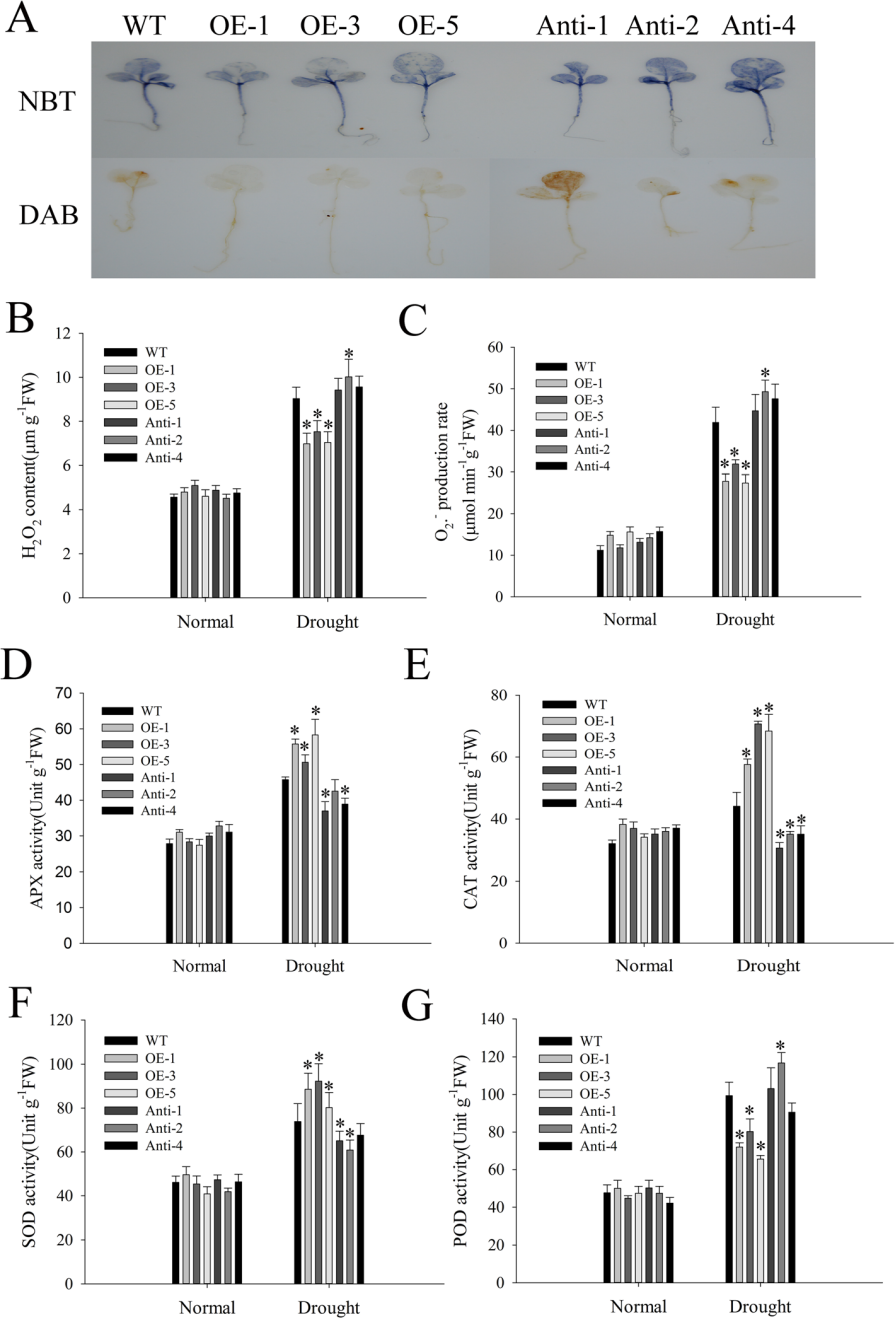
Overexpression of *NtMLP423* affects ROS production and antioxidant enzyme activities of tobacco leaves under drought stress. **a** DAB and NBT staining under drought stress. **b** H_2_O_2_ content of leaves under drought stress. **c** Oxygen free radical generation rate under drought conditions. **d**-**g** Activities of antioxidant enzymes SOD, CAT, APX, and POD under drought stress, respectively. Data points represent means ± SE (n = 3). * indicate significant difference (*P < 0.05)

**Fig. 8:**
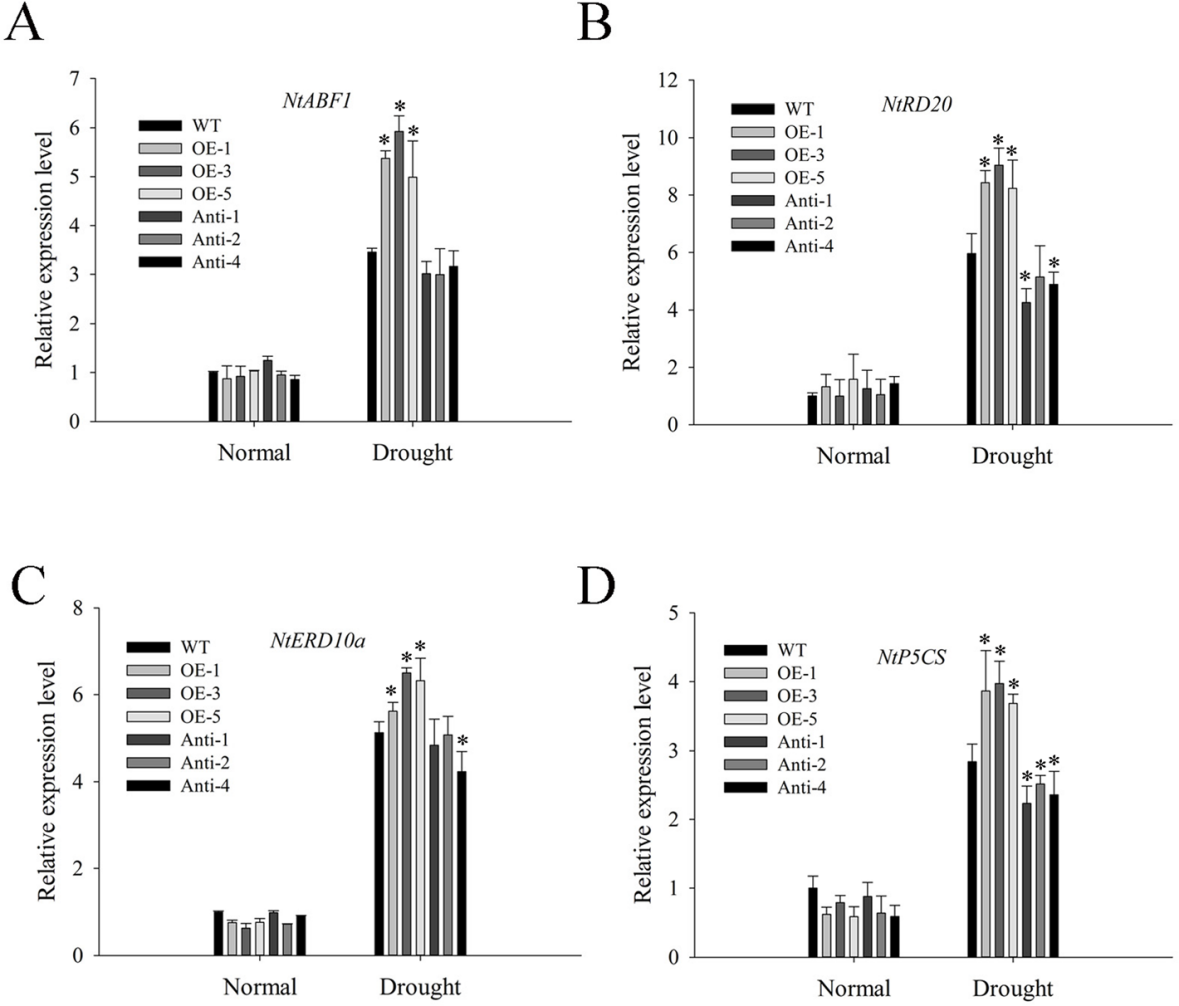
Transcript levels of stress-related genes *NtABF1, NtRD20, NtERD10a* and *NtP5CS* under drought stress. Data represent means ± SE (n = 3). * indicate significant difference relative to WT (*P < 0.05)

Drought can cause osmotic stress, which leads to ROS production [28]. We measured H_2_O_2_ and O_2_^•−^ levels under drought stress, and found that the accumulation of H_2_O_2_ and O_2_^•−^ were induced under drought stress; the content of H_2_O_2_ and O_2_^•−^ were significantly higher in antisense than in WT and overexpressed plants, with the lowest content found in overexpressed plants (Fig. 7b, c). The results of the nitro blue tetrazolium (NBT) and 3,3′-diaminobenzidine (DAB) staining were consistent with the results obtained from the quantitative analysis of H_2_O_2_ and O_2_^•−^ levels (Fig. 7a). Further, we tested the antioxidant enzyme activities and found no difference in all plants before drought stress. Additionally, the antioxidant enzyme activities of APX, CAT, SOD, and POD were tested under drought stress. The results suggested that the activities of APX, CAT, and SOD in overexpressed were significantly higher than those of WT or antisense plants; however, the POD activities were lower than those of WT (Fig. 7d-g). These results showed that overexpression of *NtMLP423* could regulate ROS levels by increasing the activities of APX, CAT and SOD. Moreover, we determined the expression of stress-related genes *NtABF1* (ABA responsive element binding factor 1), *NtRD20* (responsive to dehydration 20), *NtERD10a* (early responsive to dehydration 10) and *NtP5CS* under drought conditions by qPCR and found that overexpression of *NtMLP423* increased expression of stress-related genes under drought stress, while the expression level of antisense were lower than that of WT (Fig. 8).

### Transcription of *NtMLP423* enhanced by *NtWRKY71*

**Fig. 9:**
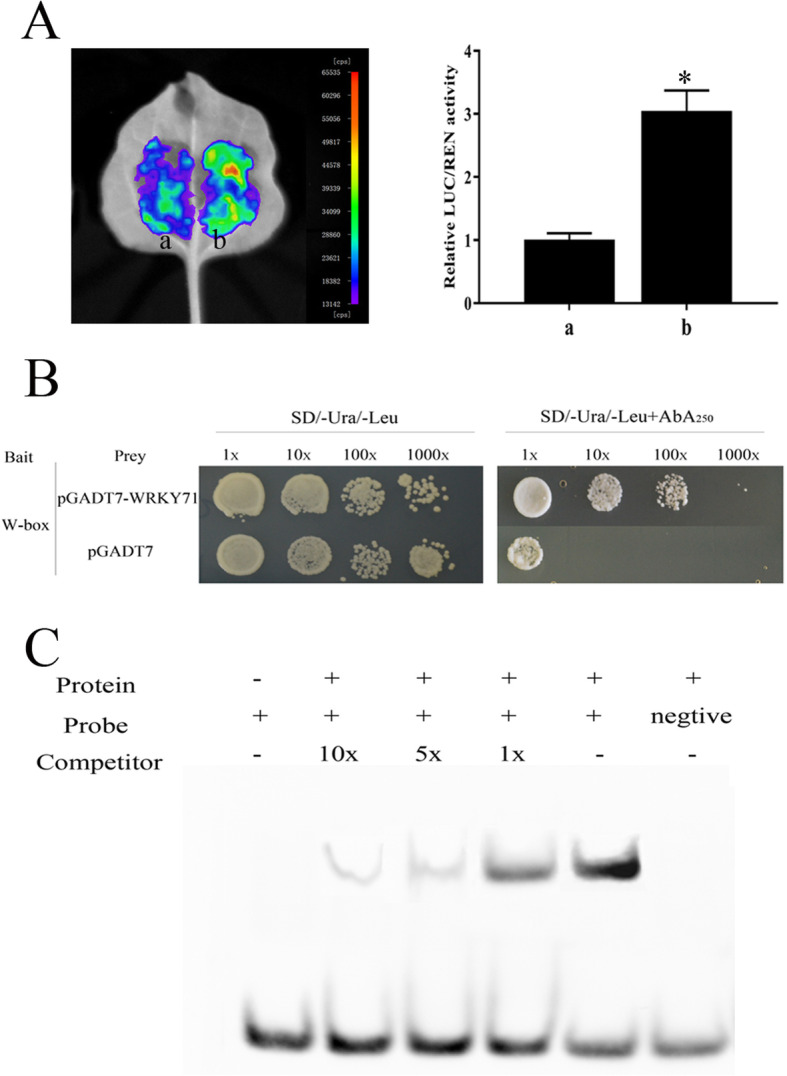
*NtWRKY71* binds to the *NtMLP423* promoter (A) *NtWRKY71* regulates the activity of the *NtMLP423* promoter. **a**: 62SK + pNtMLP423-LUC was used as the reference; **b**: NtWRKY71-62SK + pNtMLP423-LUC. The vectors were infiltrated into tobacco leaves by *Agrobacterium tumefaciens* GV3101 and then observed by firefly luciferase complementation imaging. Detection of LUC/REN activity verified that *NtWRKY71* activated the *NtMLP423* promoter. Data points represent means ± SE (n = 3). * indicated significant difference (P < 0.05). **b** Yeast one-hybrid analysis showing that *NtWRKY71* was bound to W-box in the *NtMLP423* promoter. **c** EMSA showing that the NtWRKY71 fusion protein was directly bound to the *NtMLP423* promoter on W-box in vitro

To further elucidate the molecular regulatory mechanism of *NtMLP423*, bioinformatics analysis of the *NtMLP423* promoter was performed and screened the transcription factor *NtWRKY71* for potential binding to the *NtMLP423* promoter. The firefly luciferase (Luc) complementation imaging assay were performed and observed that the fluorescence intensity increased after injection of *NtWRKY71*, indicating transactivation effects on the *NtMLP423* promoter (Fig. 9a). Yeast one-hybrid (Y1H) assays were conducted to test whether *NtWRKY71* could bind promoters of *NtMLP423*. W-box sequences were integrated into yeast cells, and the positive yeast cells were verified by gradient dilution on an SD/−Ura/−Leu-deficient medium containing aureobasidin A (AbA). The results showed that the yeast containing the bait vector pAbAi-W-box could grow normally at an AbA concentration of 250 ng/mL (Fig. 9b). Additionally, we used the DNA fragment containing the W-box sequence as a probe to validate *NtWRKY71* binding to the *NtMLP423* promoter, in EMSA experiments. The results suggested that *NtWRKY71* was directly bound to W-box in the *NtMLP423* promoter (Fig. 9c).

## Discussion

At present, research exploring the characteristics of *MLP* gene expression mainly focuses on its role in responses to abiotic and biotic stress. Studies have reported that expression of three *MLP* genes is significantly down-regulated after oxidative stress [29], and *MLP* was detected in the stem phloem juice of melon infected with the cucumber mosaic virus [30]. Additionally, the *GhMLP28* gene can respond to pathogens [31], and significant differences in *MLP* gene family expression have been shown to occur among different tissues. In our study, the *NtMLP423* gene was expressed at the highest level in leaves and roots and was significantly induced after PEG treatment (Fig. 1). The results showed that *NtMLP423* may participate in response of tobacco to drought stress. This was verified using overexpressing *NtMLP423* plants; overexpressing *NtMLP423* showed a lower wilting and a higher survival rate than WT under drought stress (Fig. 2a, 5, S5). The results indicated that *NtMLP423* positively regulated drought resistance.

Stomatal closure played an important role in adapting to drought stress, and ABA played a key role in regulating stomatal closure [32]. In this study, we showed that overexpressing transgenic plants exhibit ABA hypersensitivity, including ABA-induced inhibition of seed germination and ABA-induced promotion of stomatal closure (Figs. 2c and 3). The efficiency of water loss is related to drought stress tolerance, as water loss rapidly increases sensitivity to drought [33]. RWC of overexpressing *NtMLP423* was higher than that of WT, but the water loss rate and osmotic potential were significantly lower; this high water retention capacity benefitted transgenic plants by enhancing their drought tolerance (Fig. 5b-c, S8). Studies showed that the accumulation of ABA could improve drought resistance, thereby decreasing stomatal opening and water loss under drought conditions [34].

ABA content of overexpressing *NtMLP423* increased significantly under drought stress and was higher than that of WT (Fig. 4a); this indicates that *NtMLP423* is involved in ABA accumulation. ABA is a major signaling molecule participated in drought response, and many genes related to ABA biosynthesis have been identified [9, 35]. The NCED enzyme is important to ABA biosynthesis and has been shown to play a key role in drought stress response in Arabidopsis [36]. We analyzed expression of ABA synthetic genes (*ABA2*, *AAO3*, and *NCED3*), and found that they were upregulated (Fig. 4). The results showed that *NtMLP423* positively regulates ABA signaling and ABA accumulation by regulating ABA biosynthesis.

Photosynthesis is the basis for the survival of the biological world. Drought stress can inhibit photosynthesis in plants by destroying the photosynthetic system reaction center, which results in reduced photosynthetic efficiency. The photosynthetic pigment chlorophyll is crucial to photosynthesis in plants [37, 38]. We found that the contents of Pn and chlorophyll decreased under drought stress, with the greatest decrease recorded in the antisense plants and the least in overexpressed plants and the same results were found for the Fv/Fm ratio (Fig. 6a-c). The results indicate that overexpression of *NtMLP423* increases photosynthesis of tobacco under drought stress.

The final breakdown product of membrane lipid peroxidation is MDA, which is widely used to assess oxidative lipid damage under abiotic stress [39]. Previous studies showed that plant resistance to abiotic stress is closely related to physiological responses, mainly due to accumulation of osmoregulatory substances such as free proline [40]. Accumulation of free proline contributes to drought tolerance, thereby protecting cells from damage [41, 42]. Our results showed a much greater accumulation of free proline and a much lower accumulation of MDA in plant lines that overexpress *NtMLP423* than in WT (Fig. 6d, f). This demonstrates that overexpression of *NtMLP423* enhances the plants’ osmotic adjustment capability and alleviates damage to membrane lipids.

As direct or indirect inducers of a variety of genes which participate in stress response, ROS can serve as signaling molecules [43, 44]. Drought stress triggers the accumulation of ROS, which, in excess, could cause damage to plant cell membranes. Removal of ROS is important for plant survival under drought stress; therefore activity of antioxidant enzymes can maintain the balance of reactive oxygen metabolism and protect membrane system [45–47]. Our experimental results showed ROS accumulation under drought stress; however, ROS level of overexpressing was lower than that of WT and antisense plants (Fig. 7a-c). There is evidence indicating that ABA-enhanced drought stress tolerance is related to antioxidant enzymes [48]. The activities of SOD, CAT, and APX enzymes following drought stress were higher in *NtMLP423*-overexpressing than in other experimental groups (Fig. 7d-g). The results showed that *NtMLP423* participates in ROS-mediated drought response, and that *NtMLP423* can eliminate the negative effects of excessive ROS production under drought stress, thereby enhancing drought tolerance. *NtABF1* and *NtRD20* are drought stress responses genes. *NtP5CS is* a key gene in proline biosynthesis, whereas *NtERD10a* is a gene encoding late embryogenesis abundant proteins and is involved in regulating drought stress [24, 32, 49]. Expression levels of stress-related genes by qPCR were examined and found that overexpressed lines increased drought tolerance by upregulating stress-related genes (*NtABF1*, *NtRD20*, *NtERD10a*, and *NtP5CS*) (Fig. 8).

There is little research on the transcription factors regulating *NtMLP423*. Here, we screened a novel transcription factor, *NtWRKY71*. Y1H and EMSA assays were determined to test whether *NtWRKY71* can bind promoter of *NtMLP423*. The results indicated that *NtWRKY71* regulated the transcription of *NtMLP423* and directly bound W-box in the *NtMLP423* promoter (Fig. 9). The WRKY transcription factors comprise a large family of transcriptional regulators that play a key role in biotic stress response, ABA signaling, and physiological and biochemical processes [50, 51]. The regulation of *NtMLP423* by *NtWRKY71* was also a factor in improving drought resistance.

## Conclusions

As determined by physiological indicators and the expression levels of drought-related genes, overexpression of *NtMLP423* increased drought tolerance in Arabidopsis and tobacco. According to our results, *NtMLP423* is a gene that can positively regulate drought tolerance, is also conducive to improving the tolerance of plants to adversity and has potential applications in agricultural production.

## Methods

### Plant materials, seed germination assays, and drought treatment

*Arabidopsis thaliana* (ecotype Columbia) and *Nicotiana tabacum* cv. NC89 were used in the study. *Arabidopsis thaliana* (ecotype Columbia) plants were got from Arabidopsis Biological Resource Center (ABRC), and seeds of tobacco (*Nicotiana tabacum* cv. NC89) were stored in our laboratory. The plants seeds were sown on MS medium and germination assays were performed without and with ABA (0.5 and 1 μM) or mannitol (150 and 200 mM).

To study the effects of drought stress on Arabidopsis and tobacco, we conducted both simulated and natural-drought experiments using the seedlings obtained from the procedure outlined above. To simulated drought treatment, the roots of both Arabidopsis (8-week-old) and tobacco (4-week-old) were treated with 20% PEG for 7 days. For the natural-drought treatment, watering was discontinued for 14 days and the plant phenotypes were observed for 3 days after re-watering.

For Arabidopsis, three pots each of transgenic (OE1–1, OE4–1, and OE7–1) and WT lines were used, with four plants from the same line in each pot. For tobacco, three pots each of T2-generation overexpressing *NtMLP423* (OE-1, OE-3, and OE-5), antisense transgenic (Anti-1, Anti-2, and Anti-4) lines, and WT were used, with one plant per pot. Each pot of each treatment was considered one biological replicate. Overall, 12 and 21 pots were used for each treatment of *Arabidopsis* and tobacco, respectively.

### Generation of transgenic plants

Rapid Amplification of cDNA Ends (RACE) was used for cDNA cloning, using the GSP1 and NGSP1 primers used for the first round of RACE PCR reactions and nested PCR reactions (Table S1). The *NtMLP423* sequence was amplified with NtMLP423F and NtMLP423R primers (Table S1). The complete *NtMLP423* coding sequence was fused in a sense or antisense orientation, in PBI121 vector driven by CaMV 35S promoter. The sense and antisense expression vectors were obtained and transformed into *Agrobacterium tumefaciens* GV3101 and LBA4404. Tobacco transformation was carried out by leaf disc method, while the floral dip method was used for transformation of Arabidopsis.

### Subcellular localization of NtMLP423 protein

The NtMLP423 and green fluorescent protein (GFP) fusion expression vector was constructed and the vector was introduced into *A. tumefaciens* [52], which was then injected into leaves of *N.benthamiana* [53]. Confocal imaging was used a high-resolution confocal laser scanning microscope (Zeiss LSM880, Germany).

### Gene expression analysis

Rosette leaves of 8-week-old Arabidopsis and leaves of 4-week-old tobacco grown in soil without and with 7 days of drought treatment were sampled. Table S1 lists the primers used in real-time quantitative PCR experiments. The qRT-PCR study was conducted on the Quantitative PCR CFX96–3 detection system (Bio-Rad, USA) with three biological replicates.

### Stomatal aperture measurement

Eight-week-old Arabidopsis rosette leaves were incubated in a solution (50 mM KCl, 10 mM CaCl_2_, and 10 mM MES-KOH) for 3 h for ABA sensitivity analysis. ABA was added into incubation solution and then stomatal aperture was observed with a fluorescence microscope (AX10, Zeiss, Germany) after treatment with ABA for 1 h [33].

### Relative water content and water loss rate

Relative water content (RWC) and water loss rate were measured after Gaxiola et al. [54].

### ABA content measurements

To determine ABA content, 50 mg Arabidopsis rosette leaves grown under normal conditions and following 7 days of drought stress were sampled. ABA was extracted with 70% methanol and 0.1% formic acid and ABA content was determined according to Wang et al. [9].

### Proline contents and malondialdehyde (MDA) contents

Proline and MDA contents were performed as described by Zhang et al. [55].

### ROS contents and antioxidant enzyme activities

The H_2_O_2_ and O_2_^•−^ contents were determined according to the description of Kong et al. [56]. Activities of antioxidant enzymes were determined according to method of Wang et al. [57].

### Chlorophyll content measurement

Chlorophyll contents were performed according to the description of Wang et al. [24].

### Net photosynthetic rate and chlorophyll fluorescence parameters

Photosynthesis rate (Pn) was determined using portable photosynthesis measuring system (Ciras-3, PP Systems International, Hertfordshire, UK). Leaf maximum photochemical-efficiency was measured after steady state attainment in the dark for 30 min at 25 °C using a portable fluorometer (FMS-2, Hansatech, UK).

### Dual-luciferase assay

Dual-luciferase assay was carried out according to the description of An et al. [58]. The *NtMLP423* promoter was inserted into pGreenII0800-LUC vector, whereas *NtWRKY71* was inserted into pGreenII62-SK vector. All vectors were transformed into *A. tumefaciens* GV3101 and infiltrated into *N. benthamiana* leaves. Luminescence signals were observed by firefly luciferase complementation imaging (Xenogen, USA).

### Yeast one-hybrid assay

Interaction between the transcription factor and NtMLP423 promoter was verified by the Y1H assay as described by Zhu et al. [59].

### Electrophoretic mobility shift assays (EMSA)

EMSA experiments were carried out as described by An et al. [58].

### Statistical analysis

Data represent means ± SE of three biological replicates and SPSS software was used for statistical analysis. * indicates significantly different at *P* < 0.05, ** indicates significantly different at *P* < 0.01, relative to the WT.

## Supplementary information

**Additional file 1 Table S1.** Primers for gene amplification and qRT-PCR.

**Additional file 2 Fig. S1** (A)-(C) Subcellular localization of NtMLP423-GFP fusion protein. **Fig. S2** Relative expression of stresses reference marker genes under ROS, ABA, and drought stress. Expression level of *NtDEFL* (A), *NtABI5* (B) and *NtP5CS* (C) treated with MV, 100 μM ABA and 20% PEG, respectively. Data represent means ± SE (*n* = 3). * indicate significant difference relative to 0 h (*P < 0.05, **P < 0.01). **Fig. S3**. Identification of transgenic Arabidopsis plants. (A) PCR identification of transgenic plants, M: DL2000 marker. 1–7: OE1–1, OE2–1, OE3–1, OE4–1, OE5–1, OE6–1, and OE7–1 lines, respectively. (B) Expression level of *NtMLP423* in transgenic *Arabidopsis*. Data represent means ± SE (*n* = 3). * indicate significant difference relative to WT (*P < 0.05, **P < 0.01). **Fig. S4** *NtMLP423* participated in drought response in seed germination assay in Arabidopsis. (A) Germination of Arabidopsis seed on MS medium and with mannitol medium. (B) Statistics of germination rate under mannitol treatment. (C) Length of primary roots after mannitol treatment. (D) Statistical analysis of root length. Data represent means ± SE (n = 3). * indicate significant difference relative to WT (*P < 0.05). **Fig. S5** The *NtMLP423* gene is involved in drought stress responses in Arabidopsis. (A) Phenotypic observation of plants treated with 20% PEG for 7 days. (B) RWC in Arabidopsis under drought stress. (C) Osmotic potential in Arabidopsis leaves under drought stress. Data represent means ± SE (n = 3). * indicate significant difference relative to WT (*P < 0.05). **Fig. S6** Genes expression is involved in ABA catabolism pathway in Arabidopsis under drought stress. Data represent means ± SE (n = 3). * indicate significant difference relative to WT (*P < 0.05). **Fig. S7** Expression levels of *NtMLP423* in transgenic tobacco. (A) Expression level analysis of *NtMLP423-*overexpressing transgenic tobacco. (B) Expression level analysis of antisense transgenic tobacco. Data represent means ± SE (n = 3). * indicate significant difference (*P < 0.05, **P < 0.01). **Fig. S8** Osmotic potential of tobacco leaves under drought stress. Data represent means ± SE (n = 3). * indicate significant difference relative to WT (*P < 0.05).

**Additional file 3.**

## Data Availability

Tobacco genes sequences in this research were downloaded from National Center for Biotechnology Information (NCBI) (https://www.ncbi.nlm.nih.gov/). The primers for qRT-PCR used in this research were designed in Primer5 software and the specific primers for qRT-PCR are listed in supplementary table. The datasets used and/or analysed during the current study are available from the corresponding author on reasonable request.

## Abbreviations

ABA: Abscisic acid
GFP: Green fluorescent protein
MLP: Major latex protein
MDA: Malondialdehyde
MS: Murashige and skoog
Pn: Net photosynthetic rate
NBT: Nitro blue tetrazolium
PEG: Polyethyleneglycol
RWC: Relative water content
Fv/Fm: Variable fluorescence/maximum fluorescence
ROS: Reactive oxygen species
WT: Wild type
Y1H: Yeast one-hybrid
EMSA: Electrophoretic mobility shift assays
