# Supplementary material for: The Nicotiana tabacum L. major latex protein-like protein 423 (NtMLP423) positively regulates drought tolerance by ABA-dependent pathway
Source: BMC Plant Biol. 2020 Oct 16;20:475. doi: 10.1186/s12870-020-02690-z (PMC7565365; doi:10.1186/s12870-020-02690-z)
Supplement: Supplementary file 1 — Additional file 1 Table S1. Primers for gene amplification and qRT-PCR. [file 12870_2020_2690_MOESM1_ESM.doc]

Table S1. Primers for gene amplification and qRT-PCR.

| Primer | Forward Primer sequence (5′-3′) | Reverse Primer sequence (5′-3′) |
| --- | --- | --- |
| GSP1 | CCGACATTCCTTGCACTCTAAATTCTCT | AGTTAAGCGTGCTTGGGCGAGAGTAG A |
| NGSP1 | TCCGGTGCGTTAGTGCTGGTATGATCG | GCACTAACGCACCGGATCCCATCAGA |
| MLP423 | CGGGATCCACTACTTTTGCTCCATCCC | TCCCCCGGGCAGCAAAGAGCAAACTATG |
| qMLP423 | GGTGAAGGATCGCCATTGATTACG | GATACAGTGAGGCTGCCCTTAAAAC |
| AtActin | GGTAACATTGTGCTCAGTGGTGG | AACGACCTTAATCTTCATGCTGC |
| AtABA2 | TCCAAGCATGCTGTTCTAGG | AAATGAGCCAAAGCGAGTTT |
| AtAAO3 | TCCATCATGGACTGCTCCTTC | CGAGACACTAGCGCCAAGAAA |
| AtNCED3 | TTGATGCTCCAGATTGCTTC | GGACCCTATCACGACGACTT |
| AtBG1 | AGGCCTGCCTGACAAATTTAGC | CAGCGTTATGATTTTCACATCTA |
| AtBG2 | TGTCTGAATCAAGGAGCTTAG | AGCTCGATGTCAGAGCCAC |
| AtCYP707A1 | TGGCTCCAAAACCCAATACGT | CGAATGGCCCATACTGAATC |
| AtUGT71C5 | CATTTGAGATGGTGAAGGAGTT | TTCTATAACCTTCTTTCTCACG |
| NtActin | CATTGGCGCTGAGAGATTCC | GCAGCTTCCATTCCGATCA |
| NtABF1 | GCAATGTTCTGCTGGACAAG | TACACTCTTTCCCGCCTGTT |
| NtRD20 | GGATTTCGTGACCTTGGTTT | TAAACCGGCAATAATGGTGA |
| NtERD10A | TCTGAAGCGTGGCACTATTTCA | TCCACGGCACATCACTATAACGTAT |
| NtP5CS | ACATTAGGCGAAGAGTATTGTGTT | ACTTCACACACCCACTTACCT |
| NtDEFL | TGTTACACAAGATGCCGTGAGA | TATCGTCGCAGAAGTCGCATAA |
| NtABI5 | GAGAATGCGCAGCTAAAACA | GTGGACAACTCGGGTTCCTC |
| NtWRKY71 | CTCGAGATGATTTGGAGTAGTT | GAATTCGGATGGCTCTGGTTTA |
| pNtMLP423 | AAGCTTTGCAATGTATAACCGATGT | CCCGGGGACTATTGATTGATGGGATG |
| pAbAi-W-box | GAATTCATGATTTGGAGTAGTT | CTCGAGGGATGGCTCTGGTTTA |
