# Supplementary material for: The Nicotiana tabacum L. major latex protein-like protein 423 (NtMLP423) positively regulates drought tolerance by ABA-dependent pathway
Source: BMC Plant Biol. 2020 Oct 16;20:475. doi: 10.1186/s12870-020-02690-z (PMC7565365; doi:10.1186/s12870-020-02690-z)
Supplement: Supplementary file 2 — Additional file 2 Fig. S1 (A)-(C) Subcellular localization of NtMLP423-GFP fusion protein. Fig. S2 Relative expression of stresses reference marker genes under ROS, ABA, and drought stress. Expression level of NtDEFL (A), NtABI5 (B) and NtP5CS (C) treated with MV, 100 μM ABA and 20% PEG, respectively. Data represent means ± SE (n = 3). * indicate significant difference relative to 0 h (*P < 0.05, **P < 0.01). Fig. S3. Identification of transgenic Arabidopsis plants. (A) PCR identification of transgenic plants, M: DL2000 marker. 1–7: OE1–1, OE2–1, OE3–1, OE4–1, OE5–1, OE6–1, and OE7–1 lines, respectively. (B) Expression level of NtMLP423 in transgenic Arabidopsis. Data represent means ± SE (n = 3). * indicate significant difference relative to WT (*P < 0.05, **P < 0.01). Fig. S4 NtMLP423 participated in drought response in seed germination assay in Arabidopsis. (A) Germination of Arabidopsis seed on MS medium and with mannitol medium. (B) Statistics of germination rate under mannitol treatment. (C) Length of primary roots after mannitol treatment. (D) Statistical analysis of root length. Data represent means ± SE (n = 3). * indicate significant difference relative to WT (*P < 0.05). Fig. S5 The NtMLP423 gene is involved in drought stress responses in Arabidopsis. (A) Phenotypic observation of plants treated with 20% PEG for 7 days. (B) RWC in Arabidopsis under drought stress. (C) Osmotic potential in Arabidopsis leaves under drought stress. Data represent means ± SE (n = 3). * indicate significant difference relative to WT (*P < 0.05). Fig. S6 Genes expression is involved in ABA catabolism pathway in Arabidopsis under drought stress. Data represent means ± SE (n = 3). * indicate significant difference relative to WT (*P < 0.05). Fig. S7 Expression levels of NtMLP423 in transgenic tobacco. (A) Expression level analysis of NtMLP423-overexpressing transgenic tobacco. (B) Expression level analysis of antisense transgenic tobacco. Data represent means ± SE (n = 3). * indi [file 12870_2020_2690_MOESM2_ESM.doc]

**
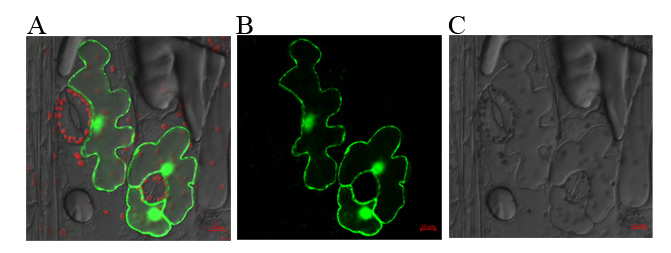
**

Fig. S1 (A)-(C) Subcellular localization of NtMLP423-GFP fusion protein.


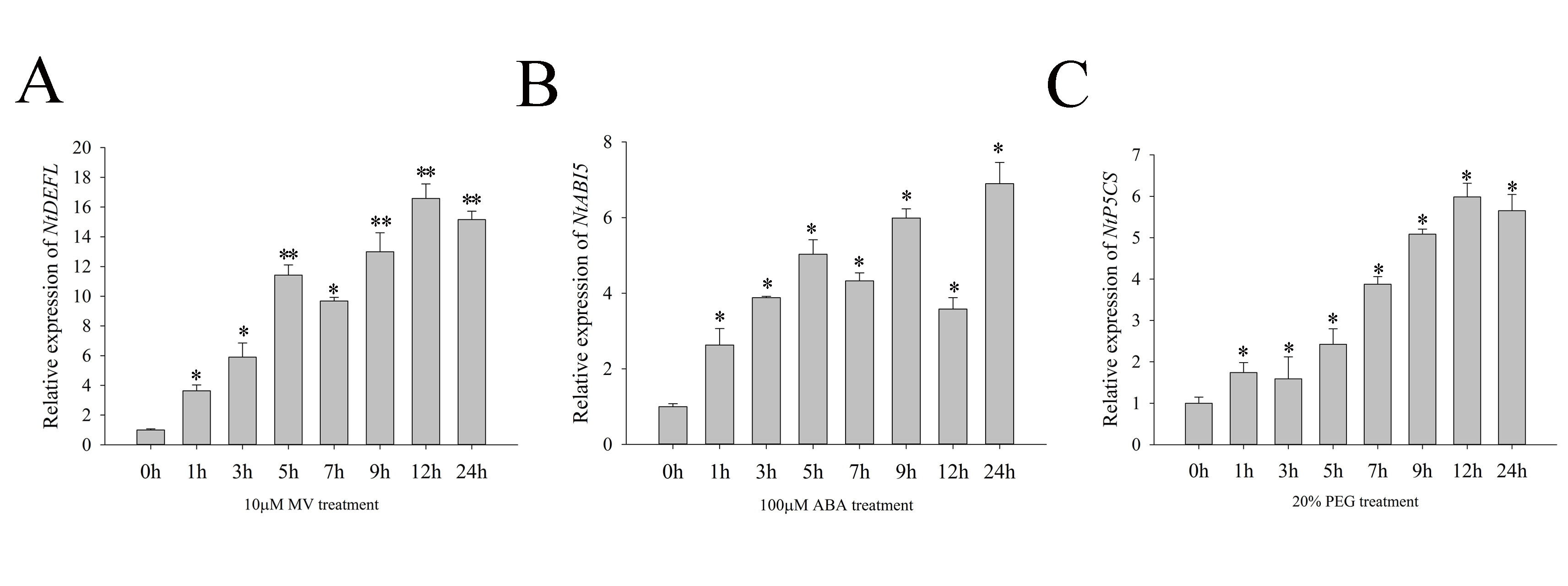


Fig. S2 Relative expression of stresses reference marker genes under ROS, ABA, and drought stress. Expression level of *NtDEFL* (A), *NtABI5* (B) and *NtP5CS* (C) treated with MV, 100μM ABA and 20% PEG, respectively. Data represent means ± SE (n = 3). * indicate significant difference relative to 0 h (*P < 0.05, **P < 0.01).


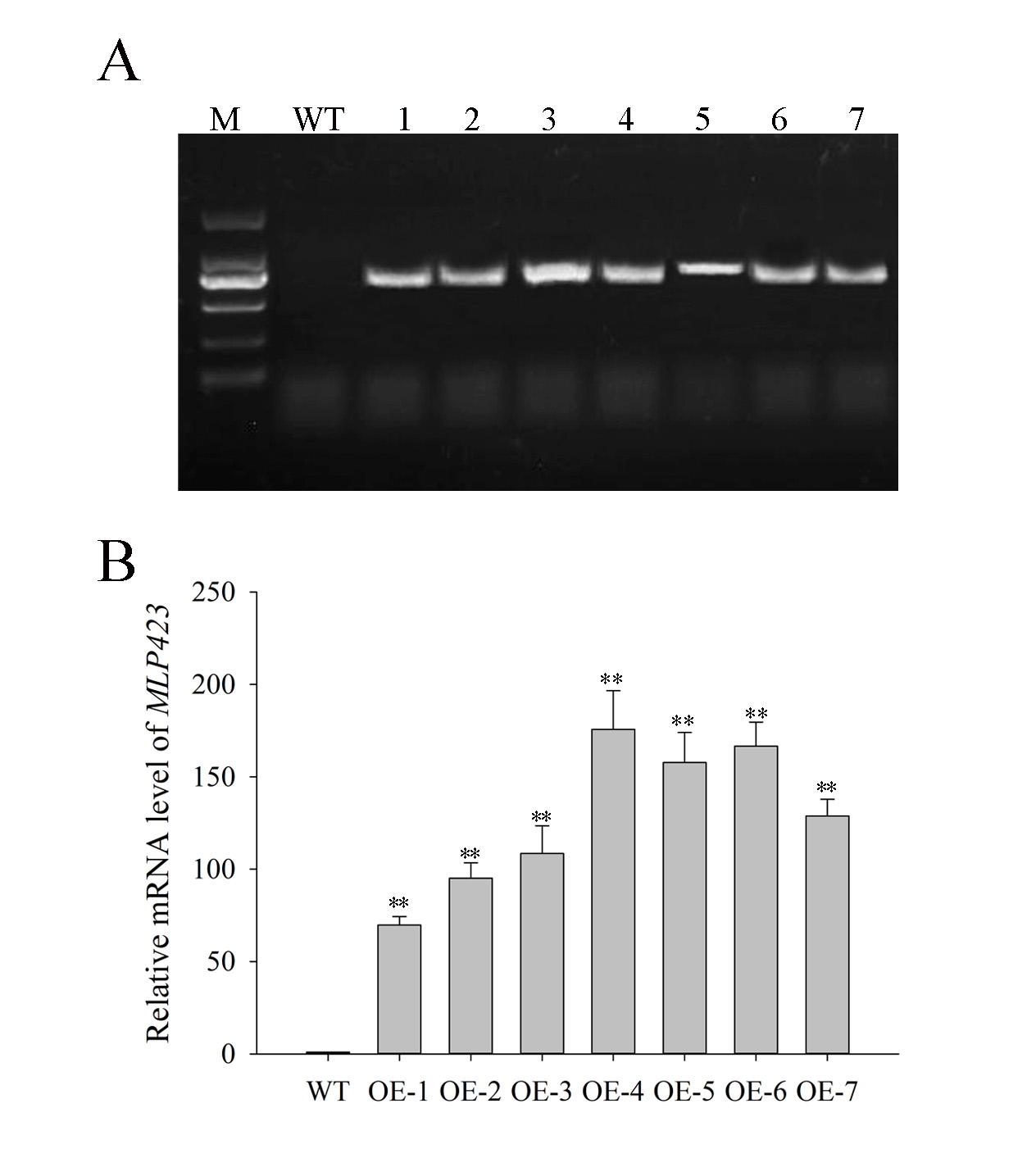


Fig. S3. Identification of transgenic Arabidopsis plants. (A) PCR identification of transgenic plants, M: DL2000 marker. 1-7: OE1-1, OE2-1, OE3-1, OE4-1, OE5-1, OE6-1, and OE7-1 lines, respectively. (B) Expression level of *NtMLP423* in transgenic *Arabidopsis*. Data represent means ± SE (n = 3). * indicate significant difference relative to WT (*P < 0.05, **P < 0.01).


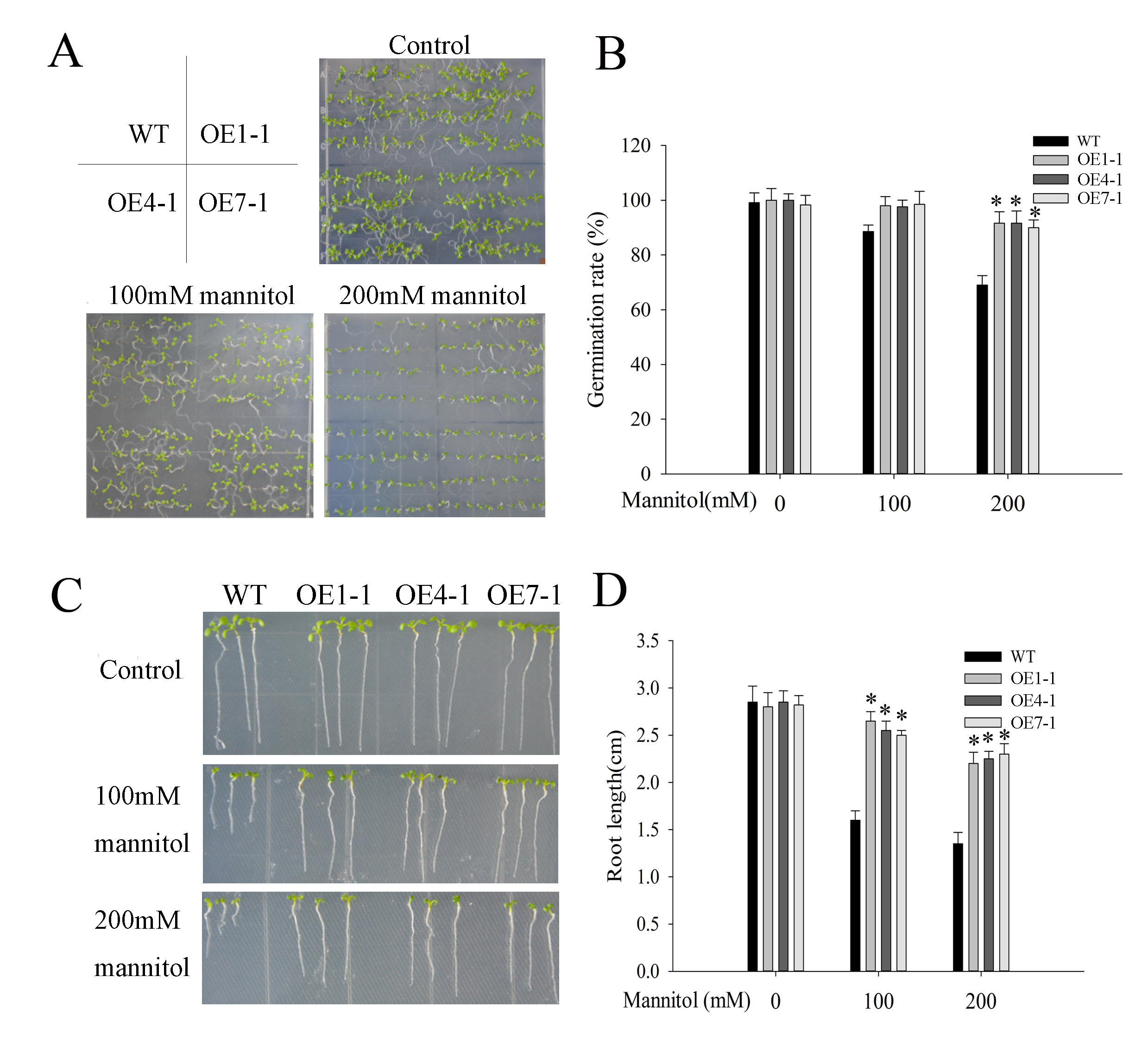


Fig. S4 *NtMLP423* participated in drought response in seed germination assay in Arabidopsis. (A) Germination of Arabidopsis seed on MS medium and with mannitol medium. (B) Statistics of germination rate under mannitol treatment. (C) Length of primary roots after mannitol treatment. (D) Statistical analysis of root length. Data represent means ± SE (n = 3). * indicate significant difference relative to WT (*P < 0.05).


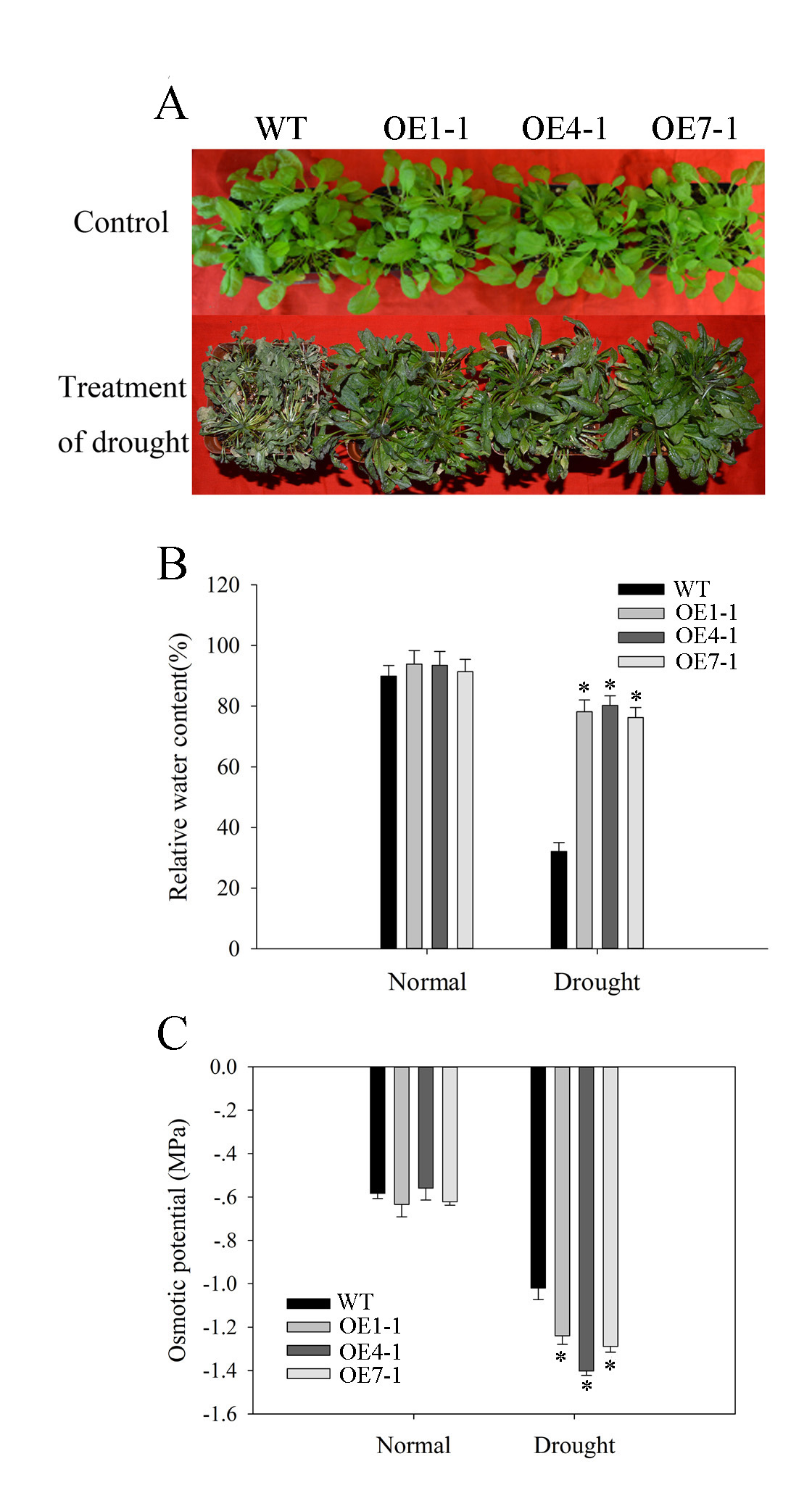


Fig. S5 The *NtMLP423* gene is involved in drought stress responses in Arabidopsis. (A) Phenotypic observation of plants treated with 20% PEG for 7 days. (B) RWC in Arabidopsis under drought stress. (C) Osmotic potential in Arabidopsis leaves under drought stress. Data represent means ± SE (n = 3). * indicate significant difference relative to WT (*P < 0.05).


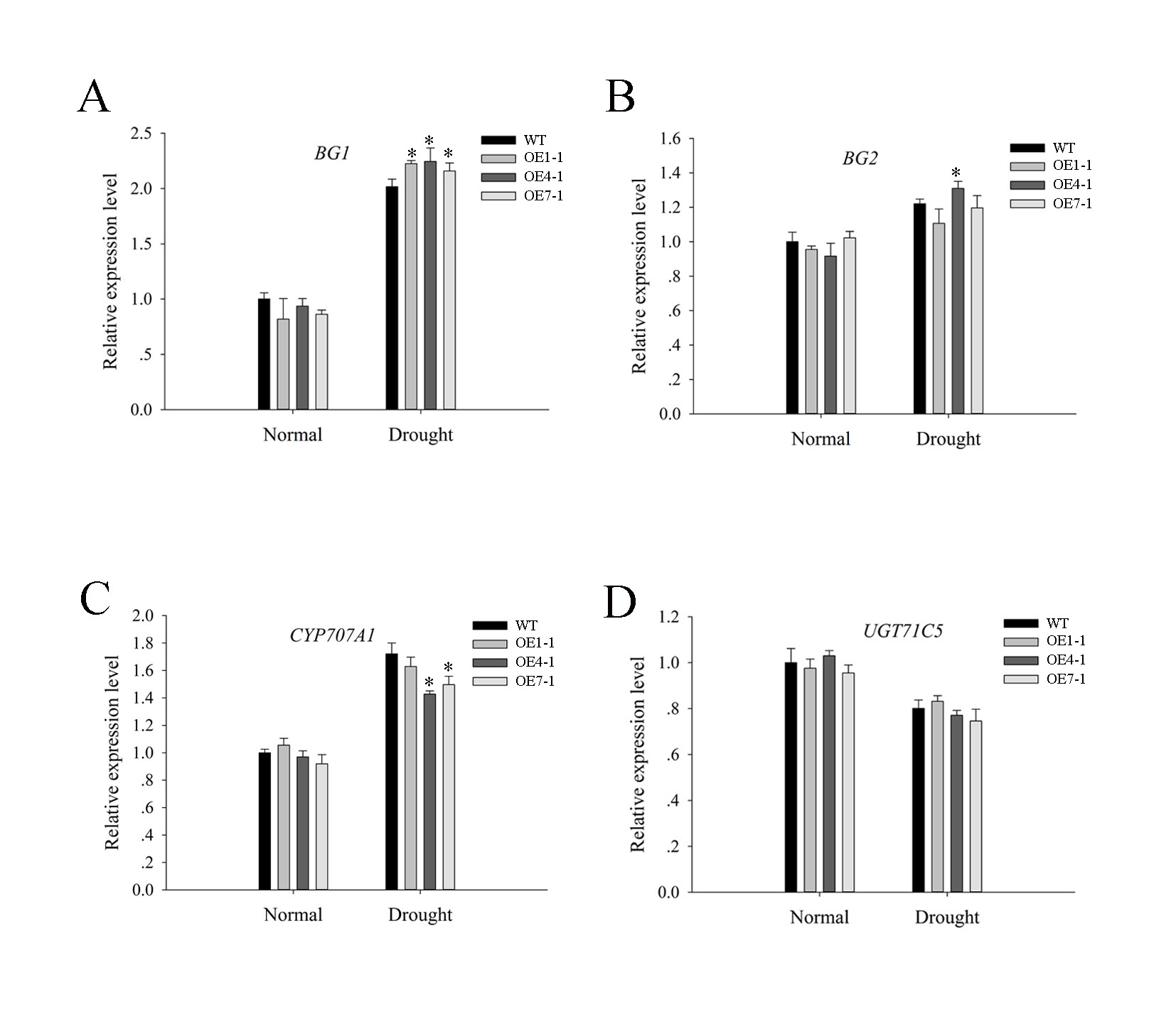


Fig. S6 Genes expression is involved in ABA catabolism pathway in Arabidopsis under drought stress. Data represent means ± SE (n = 3). * indicate significant difference relative to WT (*P < 0.05).


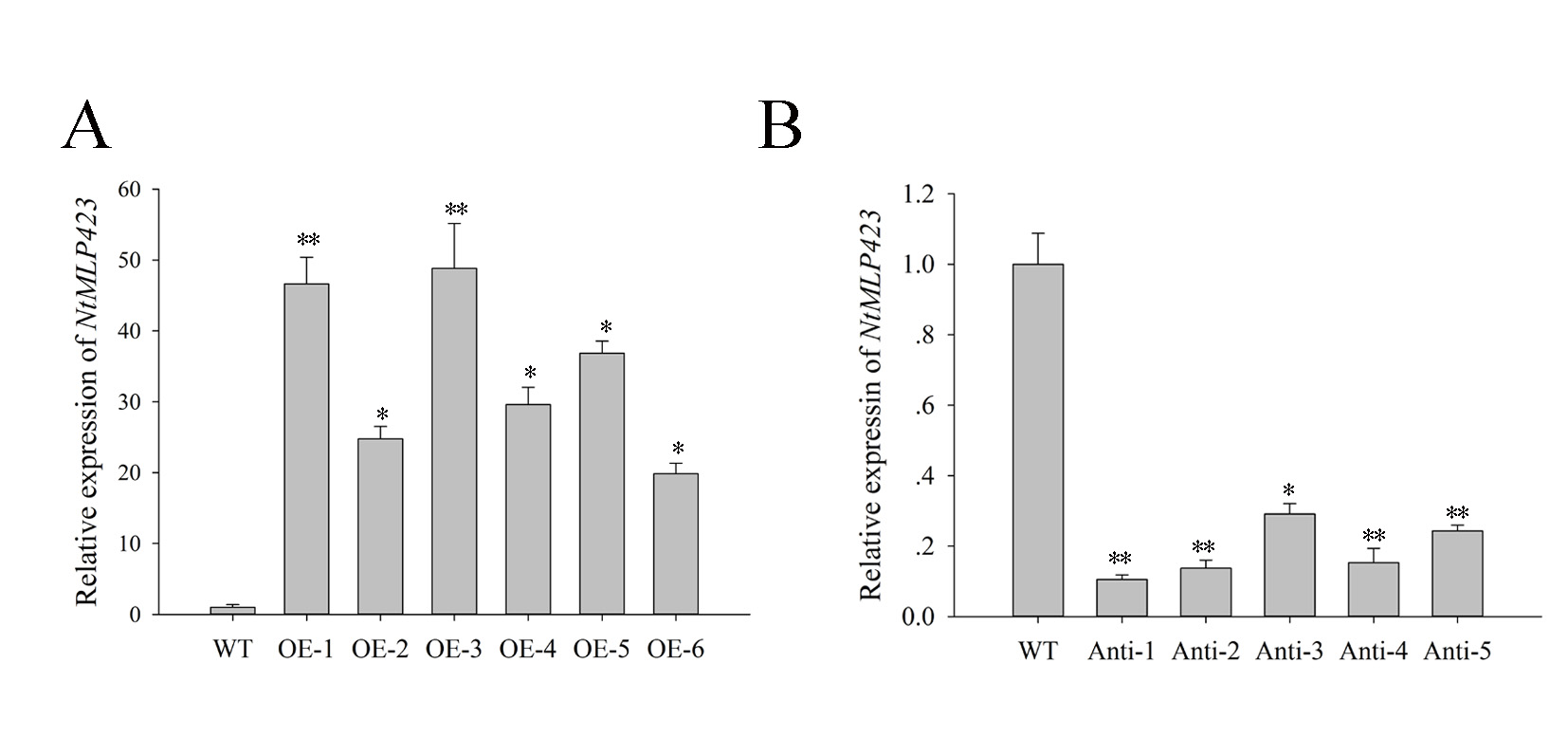


Fig. S7 Expression levels of *NtMLP423* in transgenic tobacco. (A) Expression level analysis of *NtMLP423-*overexpressing transgenic tobacco. (B) Expression level analysis of antisense transgenic tobacco. Data represent means ± SE (n = 3). * indicate significant difference (*P < 0.05, **P < 0.01).


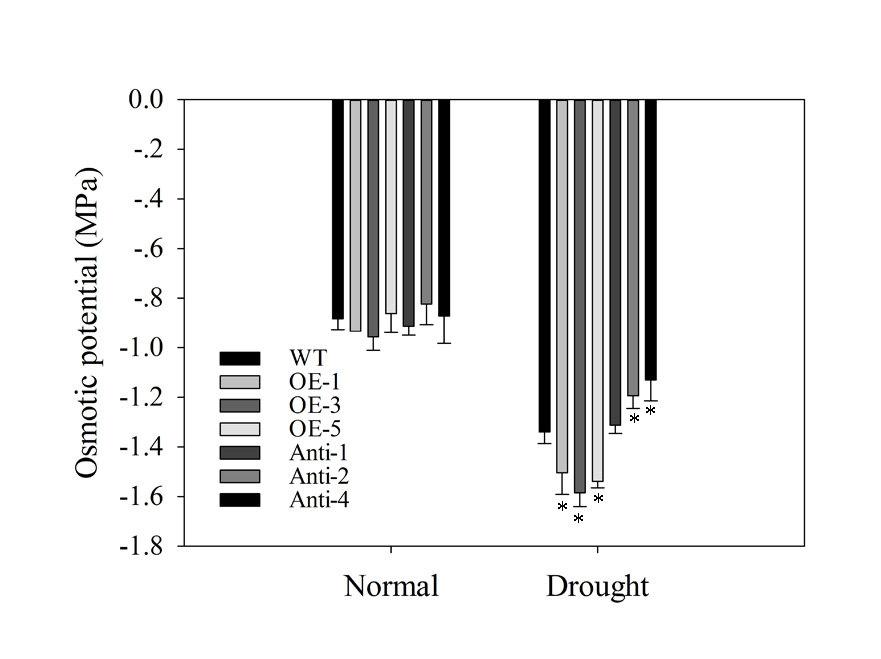


Fig. S8 Osmotic potential of tobacco leaves under drought stress. Data represent means ± SE (n = 3). * indicate significant difference relative to WT (*P < 0.05).
