# Supplementary material for: The Nicotiana tabacum L. major latex protein-like protein 423 (NtMLP423) positively regulates drought tolerance by ABA-dependent pathway
Source: BMC Plant Biol. 2020 Oct 16;20:475. doi: 10.1186/s12870-020-02690-z (PMC7565365; doi:10.1186/s12870-020-02690-z)
Supplement: Supplementary file 3 — Additional file 3. [file 12870_2020_2690_MOESM3_ESM.docx]

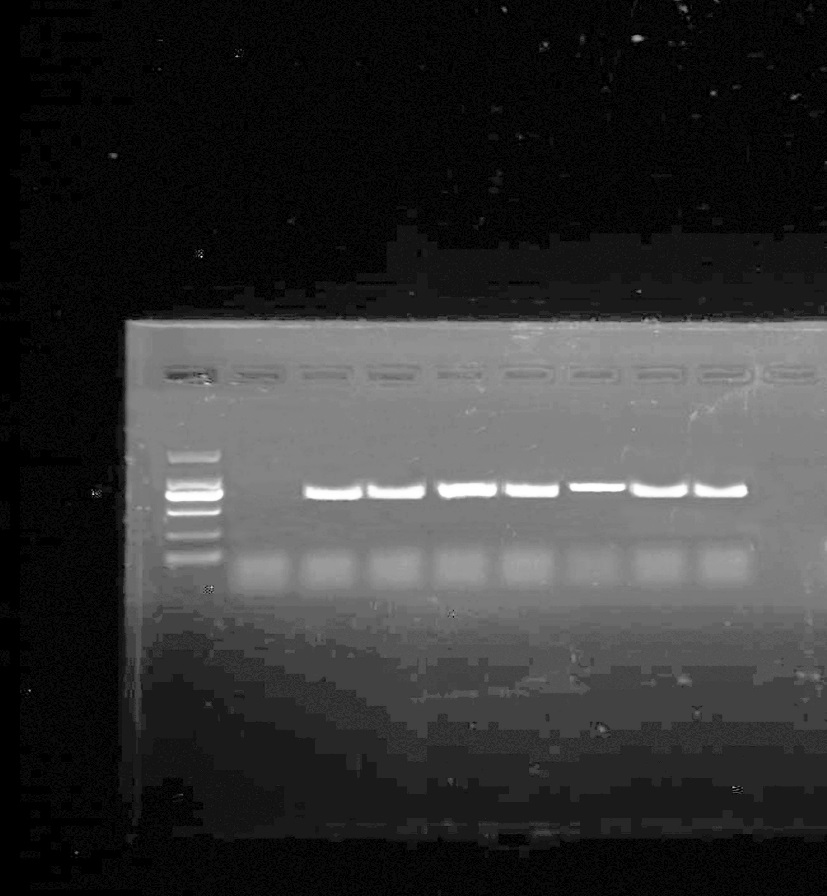


PCR identification of transgenic plants.


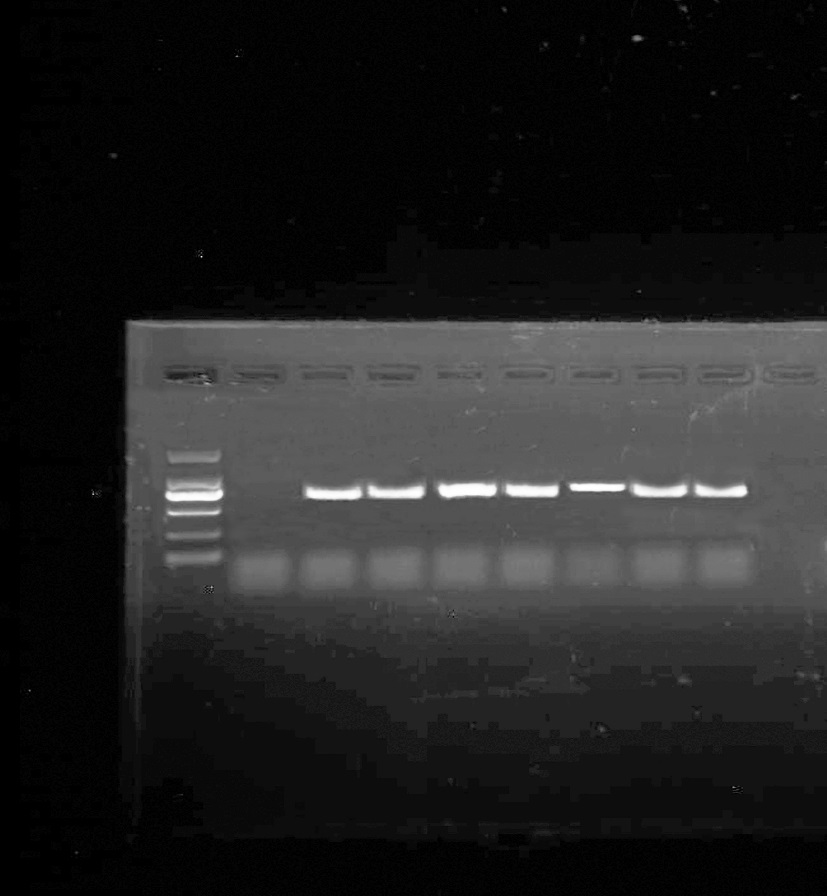


PCR identification of transgenic plants.


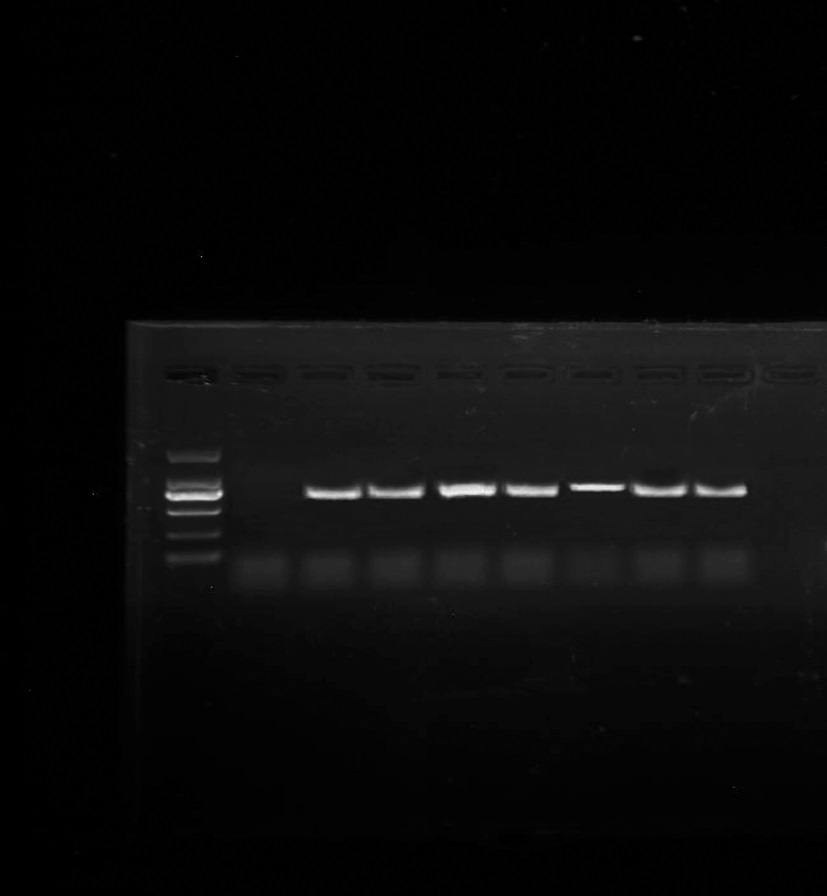


PCR identification of transgenic plants.


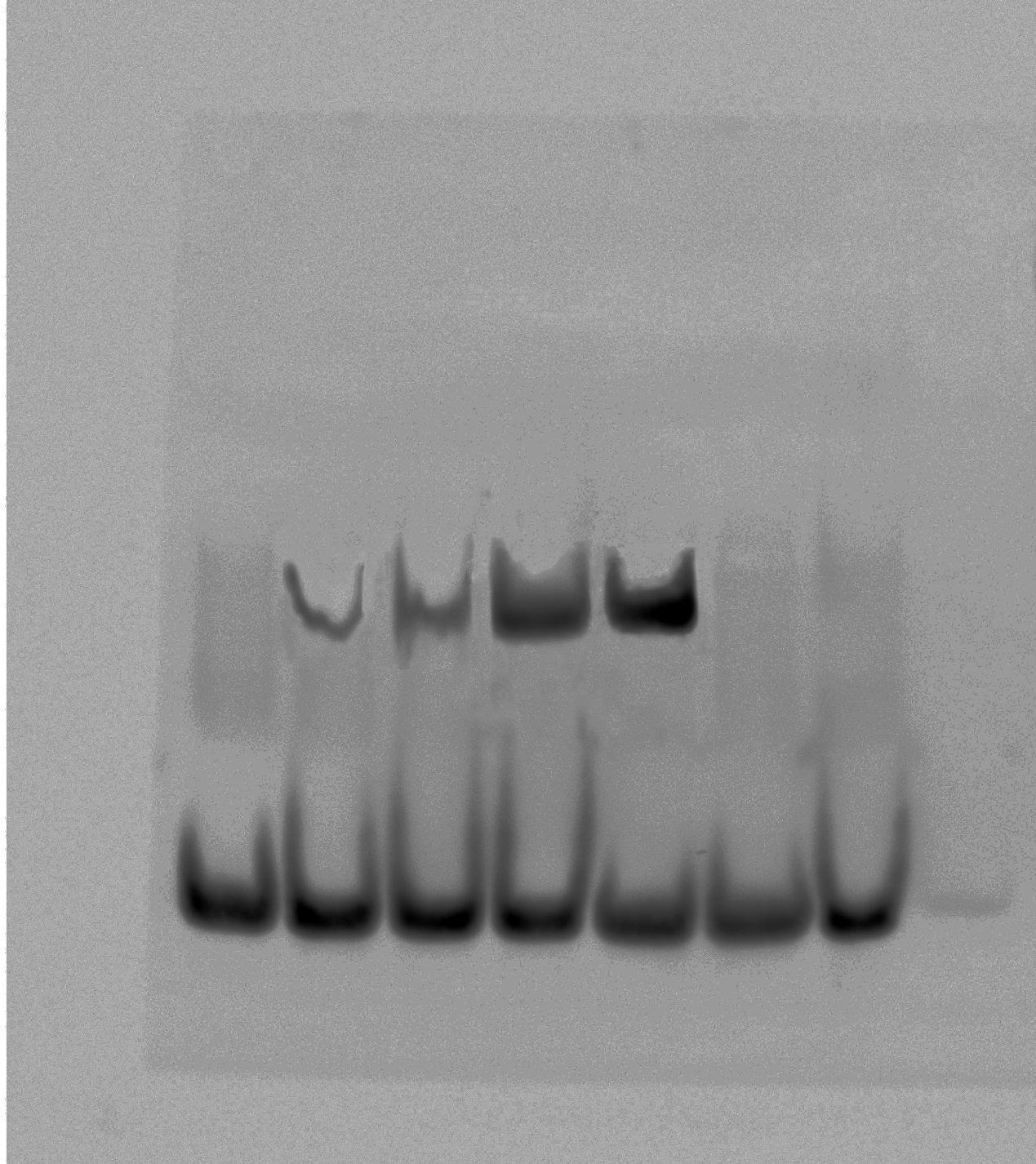


EMSA showing that NtWRKY71 fusion protein was directly bound to *NtMLP423* promoter on the W-box in vitro.


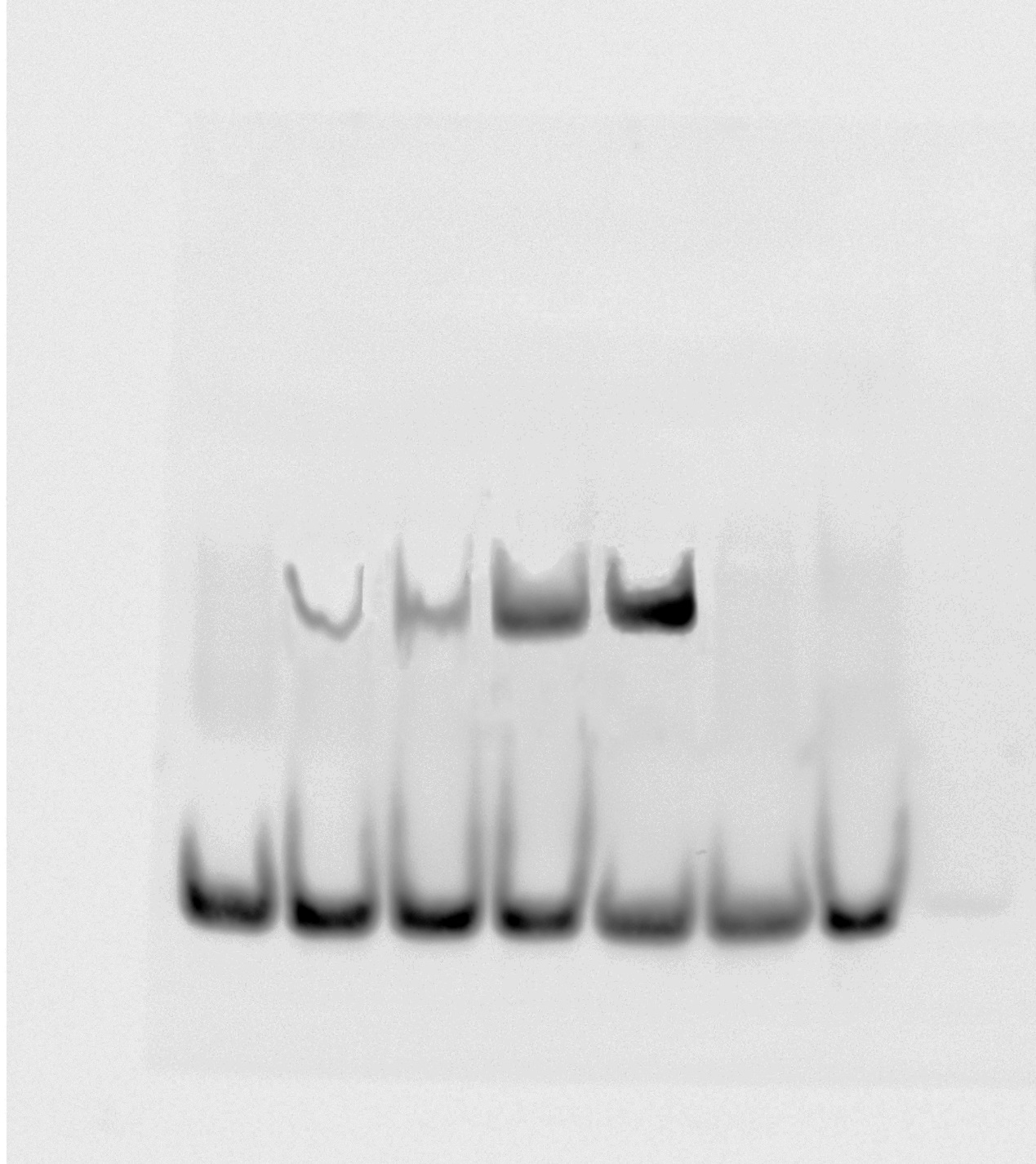


EMSA showing that NtWRKY71 fusion protein was directly bound to *NtMLP423* promoter on the W-box in vitro.


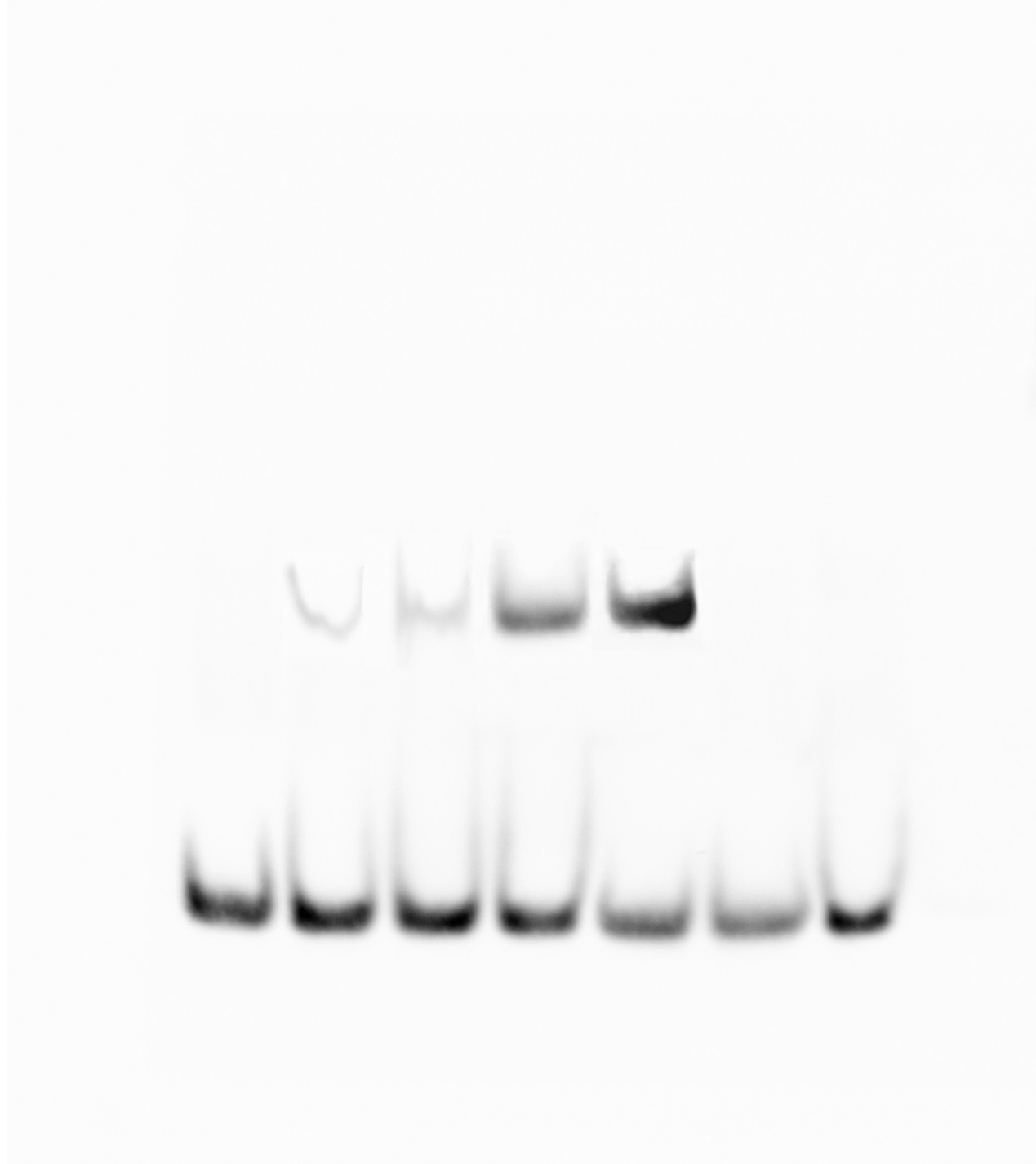


EMSA showing that NtWRKY71 fusion protein was directly bound to *NtMLP423* promoter on the W-box in vitro.
